# Dopamine-Mediated Attenuation of OECT-Based Aqueous Artificial Chemical Synapses

**DOI:** 10.1021/acsami.6c02711

**Published:** 2026-05-05

**Authors:** Haoqin Zhang, Xinzhao Xu, Waner He, Atsushi Isobe, Yunqi Liu, Yan Zhao, Tsuyoshi Michinobu

**Affiliations:** † Department of Materials Science and Engineering, Institute of Science Tokyo, 2-12-1 Ookayama, Meguro-ku, Tokyo 152-8552, Japan; ‡ Department of Materials Science, 12478 Fudan University, Shanghai 200433, P. R. China

**Keywords:** neuromorphic computation, artificial synapse, organic electrochemical transistor, floating-gate architecture, aqueous electrolyte, chemical modulation

## Abstract

Artificial synaptic electronic devices capable of chemical communication effectively emulate the functions and behaviors of biological synapses, making them indispensable for constructing neuromorphic computing systems and biomedical interfaces. Although many artificial synapses modulated by neurotransmitters and biomolecules have been developed, the attenuation of inhibitory and excitatory synaptic responses induced by these chemicals, which is indispensable for regulating biological nervous systems, has rarely been reported. Herein, we report an artificial synapse based on a floating-gate organic electrochemical transistor (OECT) in which dopamine attenuates various synaptic behaviors in an aqueous environment. By utilizing a floating-gate structure and an ionic gel as the primary electrolyte, our device exhibits characteristic inhibitory and excitatory synaptic behaviors. When dopamine is added to the secondary aqueous electrolyte, its dopaminergic oxidation further dedopes the floating gate, leading to the observed reduction in synaptic modulation. This study provides a practical approach to achieving chemically modulated synaptic behavior, contributing to the design of biointerfaced neuromorphic components.

## Graphical abstract

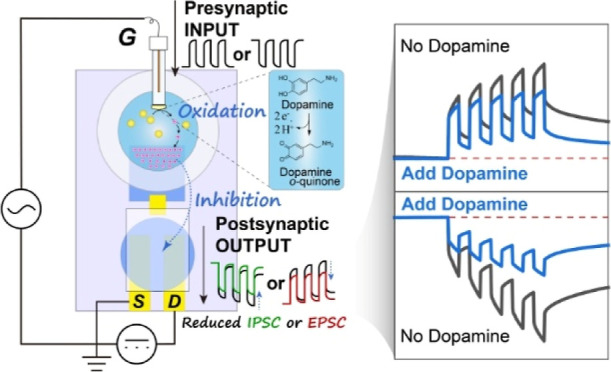

## Introduction

In the post-Moore era, the exponential growth in system scale and energy consumption demands highly parallel and energy-efficient computation.^1–4^ Whereas the conventional von Neumann architecture faces fundamental physical limitations in energy efficiency and in processing highly parallel tasks,^4–6^ biological neural systems, exemplified by the human brain, achieve remarkable energy efficiency by integrating computation and memory at the synapse.^7–9^ Consequently, brain-inspired neuromorphic computing has emerged to mimic biological neurons and synapses at the hardware level, aiming to enable low-power, fault-tolerant, and adaptive intelligent computing.^10,11^

While early neuromorphic devices utilized purely electrical signals to simulate basic Hebbian weight updates,^2,5,10–12^ they failed to capture the biochemical sophistication of biological systems. In the brain, neurotransmitters such as dopamine, acetylcholine (ACh), and γ-aminobutyric acid (GABA) not only transmit signals but also actively attenuate them to maintain neural homeostasis^12–18^ —e.g., dopamine-driven attenuation of both excitatory and inhibitory transmission in the striatum (Figure 1a).^19^ Therefore, simulating this neurotransmitter-dependent attenuation is essential for implementing biological-level gain control and enhancing energy efficiency, capabilities that purely electrical networks struggle to achieve.^20–22^ In stark contrast, most artificial synapses remain limited to physical signaling modalities, such as electrical,^23–25^ pressure,^26–28^ and optical signals,^3,29–31^ which restricts their ability to interact with chemical concentration gradients. Consequently, they lack the seamless biointegration and nonlinear dynamics inherent to biological brains.^32–35^ Therefore, to bridge the gap between artificial and biological intelligence, developing synapses capable of chemical communication is paramount, with one capability being especially critical: the ability to generate attenuation signals modulated by biomolecules such as neurotransmitters.^36^

**Figure 1:**
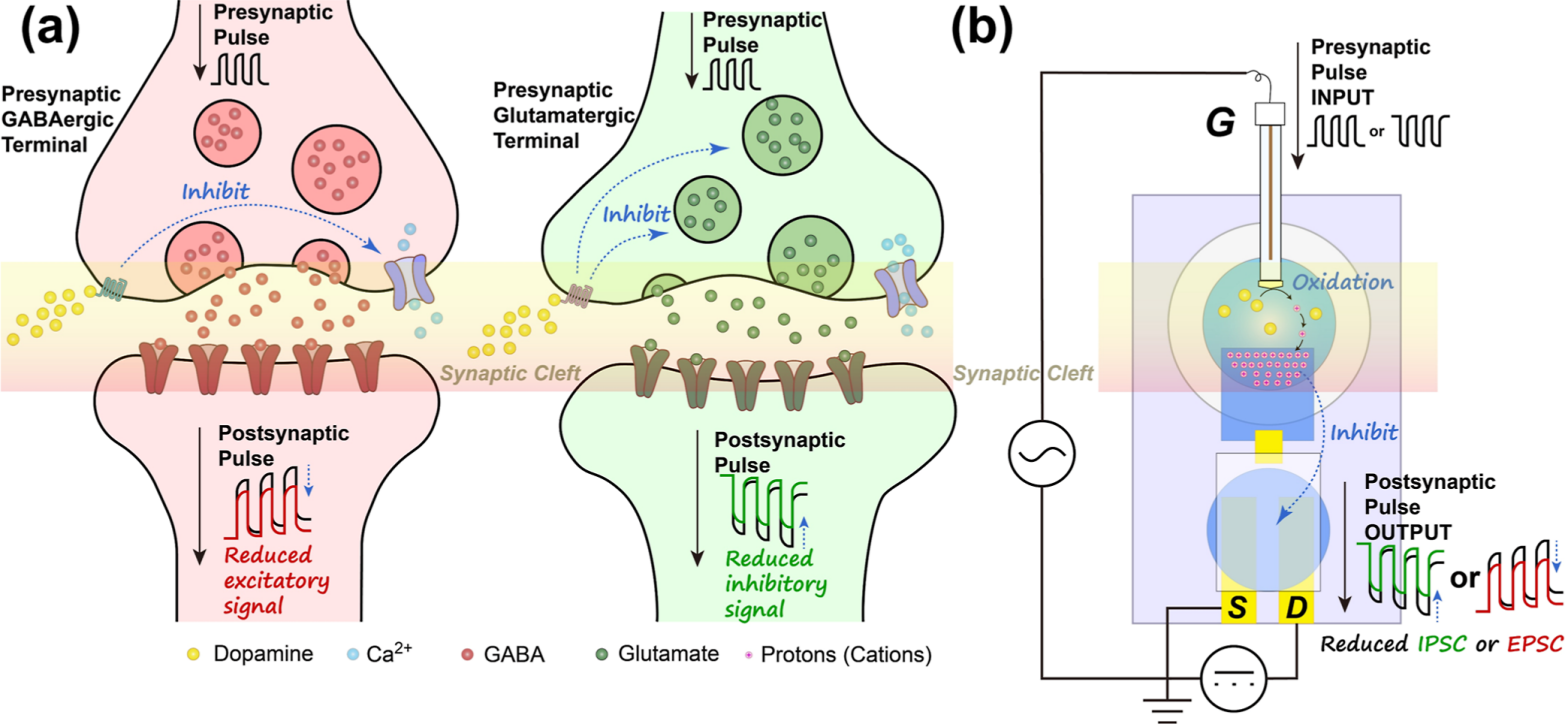
Comparison between biological synapses in the nucleus accumbens and the proposed artificial synaptic device. (a) Schematic of a biological neural network where dopamine modulates synaptic transmission, inhibiting both excitatory (GABA, left) and inhibitory (glutamate, right) pathways. (b) Schematic of the artificial synaptic device based on a floating-gate OECT. The source (S) and drain (D) electrodes are bridged by a PEDOT:PSS channel in contact with a primary ion-gel electrolyte. A floating gate connects this primary electrolyte to a secondary aqueous electrolyte containing the gate (G) electrode. Upon dopamine introduction, its oxidation in the aqueous phase induces dedoping of the floating gate, leading to the attenuation of synaptic behaviors (IPSCs and EPSCs).

To achieve artificial synapses with chemical communication, researchers have turned to organic electronics,^7,27,37–40^ where organic electrochemical transistors (OECTs) are particularly promising due to their flexibility, processability, and biocompatibility. OECTs operate via ion-electron coupling, granting them exceptionally high transconductance (*g*_m_) to amplify weak ionic signals at low voltages (<1 V). More importantly, the direct dependence of the OECT channel on ion concentration creates a natural isomorphism with the chemical weighting of biological synapses, making it inherently suitable for interfacing with biological environments.^25,34,41–49^ Building on this, numerous OECT-based devices have emulated characteristic synaptic plasticity in response to specific biochemicals. The introduction of biological neurotransmitters, e.g., dopamine and acetylcholine,^3,50–52^ or important biomolecules, e.g., glucose and lactic acid,^53,54^ has been shown to modulate typical synaptic behaviors, including short-term plasticity (STP), long-term plasticity (LTP), paired-pulse facilitation (PPF), and paired-pulse depression (PPD).^50,52,55–57^ Nevertheless, previous research on artificial chemical synapses has predominantly focused on the neurotransmitter-induced enhancement of excitatory and inhibitory signals.^5,57,58^ While chemical modulation of synaptic weight has been reported, the systematic attenuation of these behaviors—where synaptic responses decrease in correlation with increasing neurotransmitter concentration—is a fundamental biological feature that remains less explored in artificial systems.

In this work, we present an artificial synaptic device based on floating-gate OECT (Figure 1b), wherein dopamine can attenuate both excitatory postsynaptic current (EPSC) and inhibitory postsynaptic current (IPSC). To achieve this dopamine-induced attenuation, an artificial synapse based on floating-gate OECT was fabricated, incorporating poly­(3,4-ethylenedioxythiophene):poly­(styrenesulfonate) (PEDOT:PSS) as an essential component of the floating gate. PEDOT:PSS was also employed as the postsynaptic channel material, owing to its commercial availability, low operating voltage, high stability, and other advantageous properties.^53–59^ This configuration successfully recapitulates canonical synaptic plasticities, including EPSC, IPSC, PPF, and PPD. Crucially, the addition of dopamine significantly reduced both IPSCs and EPSCs as well as their associated synaptic behaviors. This neuromimetic functionality is analogous to dopamine-mediated attenuation of synaptic signals within the striatum and nucleus accumbens, where the device’s aqueous electrolyte serves as a functional analogue of the biological synaptic cleft (Figure 1). Our work specifically addresses the concentration-dependent attenuation of synaptic responses (including IPSP and EPSP). Achieving this inverse response provides a straightforward method for emulating complex inhibitory pathways, thereby enhancing the functional completeness of artificial synaptic devices for applications in neuro-prosthetics, human–machine interfaces, and neuromorphic computing systems.

## Results and Discussion

In the biological neural system, signals are transmitted from the presynaptic to the postsynaptic via synaptic clefts.^21^ More specifically, GABAergic synapses and glutamatergic synapses induce inhibitory and excitatory postsynaptic signals, respectively (Figure 1a).^19,60^ To mimic the aforementioned phenomenon, an OECT-based artificial synaptic device with a floating gate structure is fabricated (Figures 1b, 2a, and S1a). First, chromium and gold (Cr/Au) metals are sequentially deposited on the surface of the glass substrate through a patterned shadow mask, forming the source and drain electrodes as well as part of the floating gate electrode. The floating gate structure is employed because the semiconductor channel is isolated from direct contact with the aqueous environment and biomolecules, thereby protecting the transistor component of the device while also offering greater flexibility for device modification.^61^ For the postsynaptic channel, a PEDOT:PSS thin film was formed between the source and drain electrodes by spin-coating a preformed PEDOT:PSS solution. At the same time, an extra amount of PEDOT:PSS solution was spin-coated on the floating gate electrode to form another PEDOT:PSS thin film, which constitutes the remaining integral part of the floating gate electrode. To complete the floating gate structure, an ion gel/poly­(vinylidene fluoride-*co*-hexafluoropropylene) (P­(VDF-HFP)) thin film was employed as the primary electrolyte, directly contacting both the PEDOT:PSS postsynaptic channel and the Cr/Au floating gate electrode. The secondary electrolyte of the device was aqueous, allowing biomolecules, such as dopamine, to bind to the PEDOT:PSS floating gate and modulate the electrical output. Finally, a silver/silver chloride (Ag/AgCl) reference electrode was inserted into the secondary aqueous electrolyte, serving as the control gate electrode. The structural integrity and chemical consistency of this multilayer architecture were validated by scanning electron microscopy (SEM, Figure S1b,c) and Fourier-transform infrared spectroscopy (FTIR, Figure S1d), which revealed a uniform, crack-free morphology at the active interfaces and preserved the characteristic chemical signatures of PEDOT:PSS.

**Figure 2:**
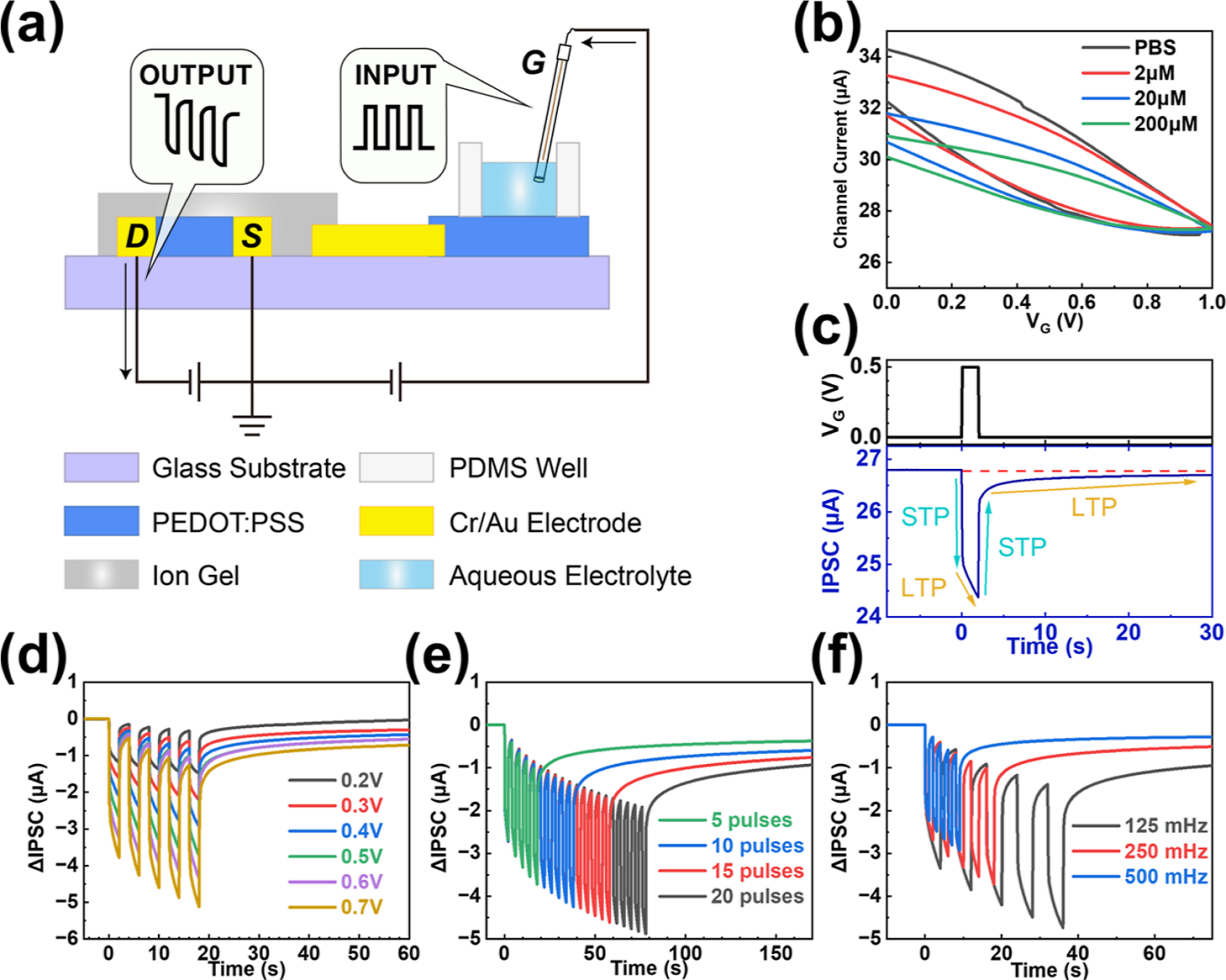
Electrochemical properties and synaptic characteristics of the PEDOT:PSS-based device under positive gate voltage pulses. (a) Schematic illustration of the device operation mechanism under positive gate bias. (b) Transfer characteristics measured in the aqueous electrolyte. (c) Synaptic response triggered by a single gate voltage pulse (*V*_G_ = +0.5 V, *t* = 2 s). (d–f) Synaptic plasticity and memory effects modulated by varying pulse parameters: (d) varying pulse amplitudes (+0.2 V to +0.7 V, fixed duration 2 s, 5 pulses); (e) varying number of pulses (5 to 20 pulses, fixed +0.5 V, 2 s); (f) varying pulse durations (1, 2, and 4 s, fixed +0.5 V, 5 pulses).

We first studied the electrochemical properties of the device by sweeping the gate voltage between 0 and +1 V for five cycles while maintaining a fixed drain-source voltage of +0.1 V. The resulting transfer curve revealed that increasing the gate voltage leads to a decrease in channel current, which is attributed to the electrochemical dedoping effect of the PEDOT:PSS channel (Figures 2b and S2). It should be noted that the transfer curves of the device exhibited typical hysteresis behaviors, indicating its ability to emulate synaptic weight modulation regulated by presynaptic electrical signals.^25,28^ In biological neuron systems, the synaptic weight of a synapse can be modulated by presynaptic electrical pulses. In this regard, the synaptic behavior (IPSC and EPSC) of the device was systematically investigated by applying positive and negative voltage pulses (*V*_G_, *t*) to the Ag/AgCl gate electrode.

Upon applying a single positive pulse (*V*_G_ = +0.5 V, *t* = 2 s) to the gate electrode of the device (Figure 2a,c), an electric field was established across the gate circuit, causing cations in the ion gel to enter the channel and extract holes from the PEDOT backbone of the PEDOT:PSS channel. As a result, the channel current decreased immediately (<50 ms) when the gate voltage increased, resulting in an IPSC that represents the STP of the synaptic device. While *V*_G_ was maintained at +0.5 V, the bulky ions in the ion gel induced LTP of the channel conductance. The injection of ions and the dedoping of the channel continued, resulting in a continuous decrease in the channel current. After the voltage pulse was removed, the channel conductance recovered, but did not immediately return to its initial value due to the LTP effect of IPSC.

A key feature of biological synapses is that repeated stimulation can further enhance changes in synaptic weight, which is crucial for learning and memory in the biological nervous system.^62,63^ To emulate this behavior, five consecutive positive voltage pulses (*V*_G_ = +0.5 V, *t* = 2 s) were applied to the gate electrode of our device (Figure S3a, top). Due to the LTP of the device under a gate voltage pulse, it showed prolonged retention of the depressed state (Figure 2c). Consequently, under consecutive gate voltage pulses (Figure S3a, top), the device underwent cumulative dedoping, manifested as a stepwise reduction in the channel current and a corresponding memory effect of the IPSC (Figure S3a, bottom). The depression behavior and memory effect were further enhanced by increasing the pulse amplitude (Figure 2d, *t* = 2 s, 5 pulses), the number of pulse series (Figure 2e, *V*_G_ = +0.5 V, *t* = 2 s), or the pulse duration (Figure 2f, *V*_G_ = +0.5 V, 5 pulses). The above phenomena resemble spike intensity-dependent plasticity (SIDP), spike number-dependent plasticity (SNDP), and spike frequency-dependent plasticity (SFDP) in biological neuronal systems, respectively.

Similar to IPSC, the neuromorphic device also generated an EPSC when a negative gate voltage pulse was applied (Figure 3a). When a single negative pulse (*V*_G_ = −0.5 V, *t* = 2 s) was applied to the gate electrode (Figure 3b), an electric field opposite to that generated under a positive gate voltage was established across the gate circuit, driving cations out of the channel and resulting in channel doping. Consequently, the channel current of the synaptic device surged immediately, exhibiting an STP effect in the EPSC. As *V*_G_ was maintained at −0.5 V, channel doping continued, resulting in an LTP effect in the EPSC. After the removal of the voltage pulse, similar STP and LTP effects were also observed. Likewise, applying consecutive negative gate voltage pulses (Figure S3b, top, 5 pulses, *V*_G_ = −0.5 V, *t* = 2 s) induced cumulative doping, manifested as an increase in channel current and a corresponding EPSC memory effect (Figure S3b, bottom). SIDP (Figure 3c), SNDP (Figure 3d), and SFDP (Figure 3e) in excitatory biological synapses can also be mimicked by modulating the amplitude, number, and duration of the negative gate voltage pulses, respectively.

**Figure 3:**
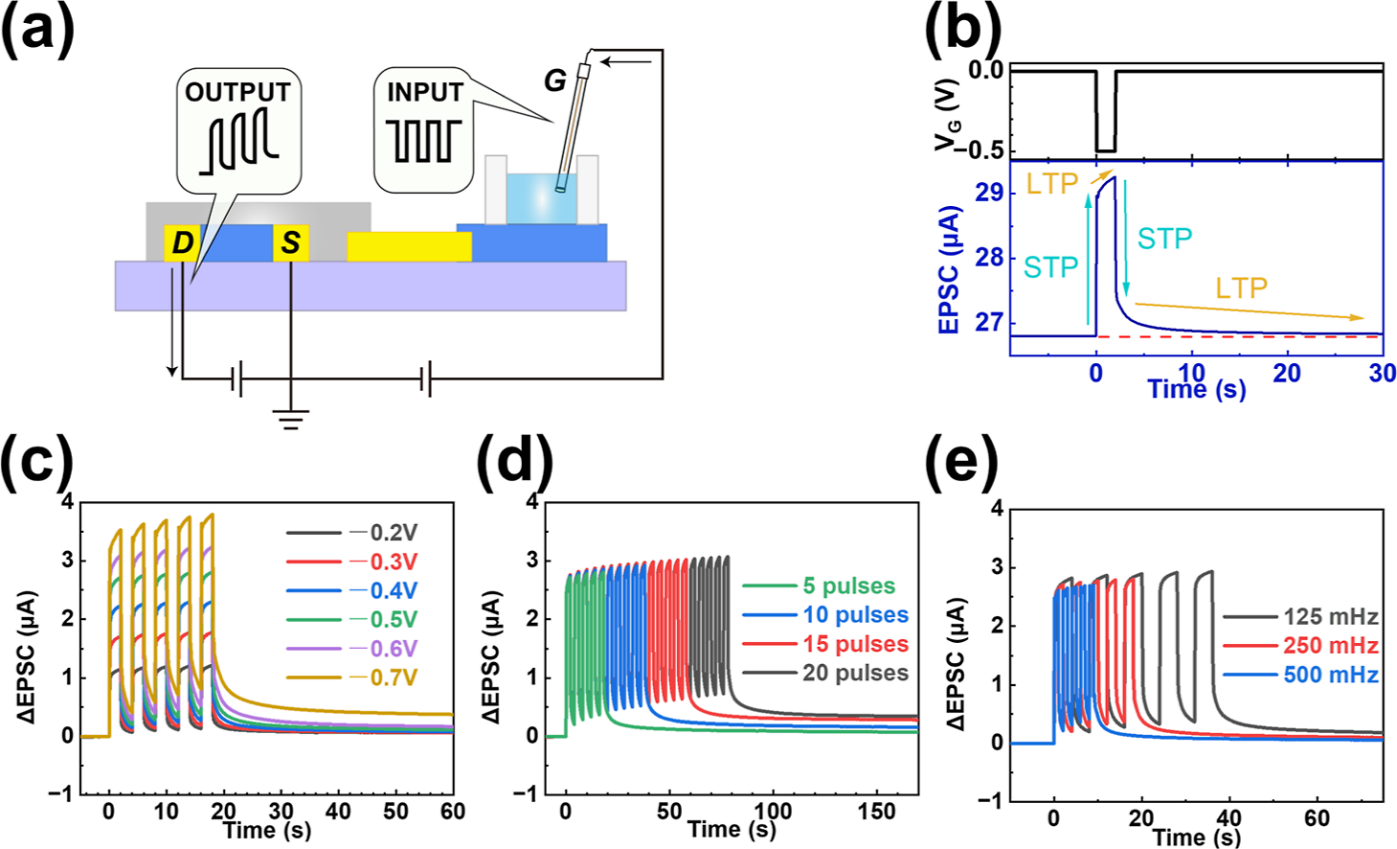
Electrochemical and synaptic characteristics of the PEDOT:PSS-based device under negative gate voltage pulses. (a) Schematic illustration of the device operation mechanism under negative gate bias. (b) Synaptic response triggered by a single negative gate pulse (*V*_G_ = −0.5 V, *t* = 2 s). (c–e) Synaptic plasticity and memory effects modulated by varying pulse parameters: (c) varying pulse amplitudes (−0.2 V to −0.7 V, fixed duration 2 s, 5 pulses); (d) varying number of pulses (5 to 20 pulses, fixed −0.5 V, 2 s); (e) varying pulse durations (1, 2, and 4 s, fixed −0.5 V, 5 pulses).

In striatal and accumbal synapses, both excitatory and inhibitory postsynaptic signals are primarily inhibited by synaptic cleft neurotransmitters such as dopamine, a process essential for normal nervous system function. Notably, in our device, the addition of dopamine induced doping of the PEDOT:PSS postsynaptic channel, leading to an increase in channel current (Figure S4). This result indicates that the aforementioned synaptic behaviors, including inhibitory and excitatory responses, can also be modulated by dopamine (Figure 4a). By examining the device transfer characteristics before and after the addition of dopamine at different concentrations (Figure 4b), we observed that the drain current shift (Δ*I*_DS_) at a fixed gate voltage decreased upon dopamine addition compared with the Δ*I*_DS_ in the absence of dopamine, which continued to shrink with increasing dopamine concentration. These results indicate that dopamine exposure reduces the hysteresis observed in the transfer curve, with higher dopamine concentrations further diminishing the hysteresis, suggesting that dopamine addition may attenuate the synaptic behavior of the device.

**Figure 4:**
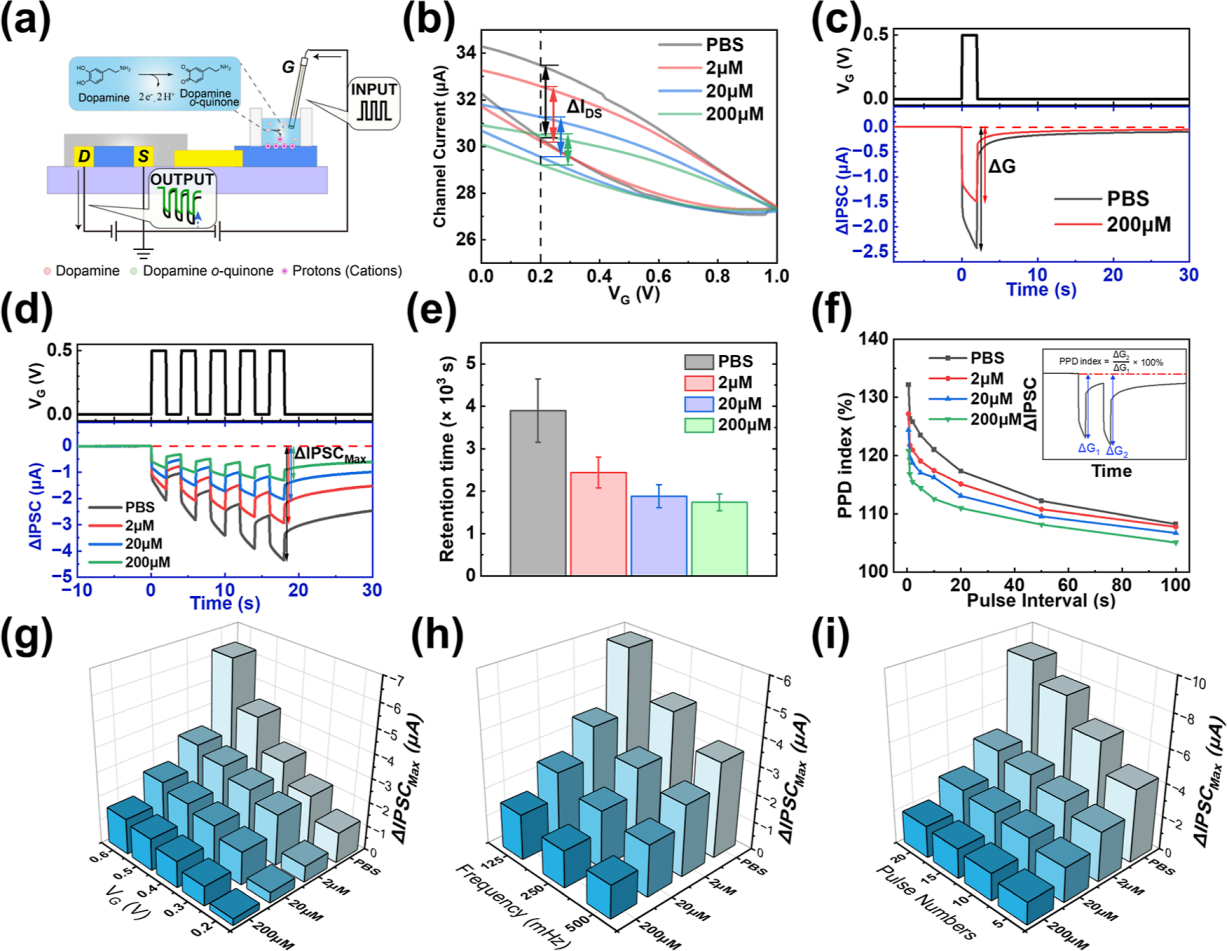
Dopamine-modulated synaptic responses under positive gate pulses. (a) Schematic illustration of the attenuation of synaptic responses by dopamine under positive gate bias. (b) Transfer characteristics measured and the change of drain current shift (Δ*I*_DS_) at a fixed gate voltage (e.g., 0.2 V) at various dopamine concentrations. (c) Synaptic response to a single gate pulse (*V*_G_ = +0.5 V, *t* = 2 s) measured without dopamine (black) and with 200 μM dopamine (red). (d) Synaptic responses and memory effects under five consecutive pulses (*V*_G_ = +0.5 V, *t* = 2 s) at varying dopamine concentrations (2 μM, 20 μM, and 200 μM). (e) The retention times (with error bars) of our device under identical 5 voltage pulses (*V*_G_ = +0.5 V, *t* = 2 s) at different dopamine concentrations. (f) PPD indices as a function of pulse interval (Δ*t*) and dopamine concentration. Inset: definition of the PPD index. (g–i) Dependence of (g) SIDP, (h) SNDP, and (i) SFDP of IPSCs on dopamine concentration.

To mimic the reduction of synaptic weight (both inhibitory and excitatory) in the biological nucleus accumbens, 200 μM of dopamine was introduced into the secondary aqueous electrolyte (Figure 4a), and the same electrical measurements were performed as those conducted in the absence of dopamine. To be specific, the same single positive voltage pulse (*V*_G_ = +0.5 V, *t* = 2 s) was applied both before and after the introduction of 200 μM dopamine into the secondary aqueous electrolyte (Figure 4c, top). Before the introduction of dopamine, the IPSC exhibited obvious STP and LTP (the black line in Figure 4d, bottom). Upon the addition of dopamine to the secondary electrolyte, both STP and LTP behaviors of the device were significantly suppressed, as evidenced by the pronounced decrease in the IPSC change observed both immediately after *V*_G_ increased to +0.5 V and while *V*_G_ was maintained at +0.5 V (red line in Figure 4c, bottom). This behavior corresponds to dopamine-induced inhibition of inhibitory postsynaptic signaling in glutamatergic synapses of the biological nucleus accumbens. Typically, the introduction of 200 μM dopamine reduced the change (Δ*G*) of IPSC after maintaining *V*_G_ at +0.5 V for 2 s to approximately 60% of its original value (Figure 4c). Similarly, when five successive positive gate voltage pulses were applied (Figure 4d, top), the LTP-related dedoping memory effect induced by multiple stimulations was markedly diminished by dopamine addition (black and green lines in Figure 4d, bottom). Similar behavior was also observed under negative gate voltage pulses (Figure 5a). For either single or five consecutive negative gate voltage pulses (Figure 5b,c), the addition of dopamine to the secondary electrolyte suppressed the STP and LTP behaviors, as well as the associated EPSC memory effects, although to a lesser extent than for IPSCs. This phenomenon is analogous to the dopamine-induced attenuation of excitatory postsynaptic signaling in GABAergic synapses of the nucleus accumbens.

**Figure 5:**
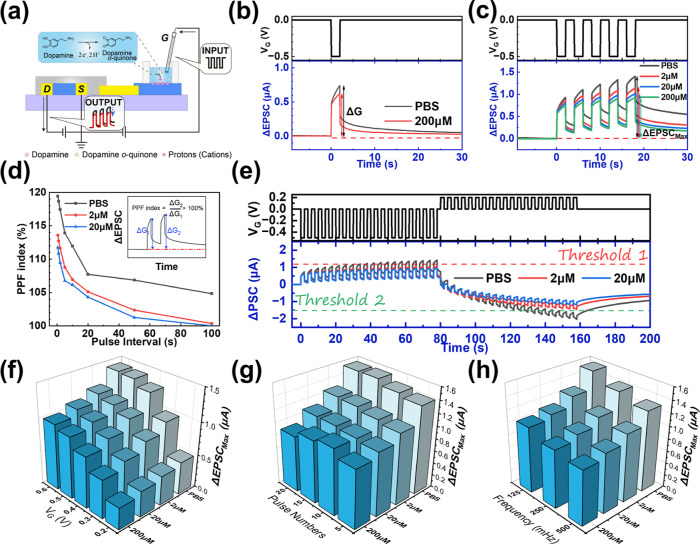
Dopamine-modulated synaptic responses under negative gate pulses. (a) Schematic illustration of the attenuation of synaptic responses by dopamine under negative gate bias. (b) Synaptic response to a single gate pulse (*V*_G_ = −0.5 V, *t* = 2 s) measured without dopamine (black) and with 200 μM dopamine (red). (c) Synaptic responses and memory effects under five consecutive pulses (*V*_G_ = −0.5 V, *t* = 2 s) at varying dopamine concentrations (2 μM, 20 μM, and 200 μM). (d) PPF indices as a function of pulse interval (Δ*t*) and dopamine concentration. Inset: Definition of the PPF index. (e) Emulation of physiological learning and forgetting processes under alternating negative and positive pulses (20 cycles). The curves at the bottom demonstrate the inhibitory modulation of learning or forgetting behaviors by varying dopamine concentrations (colored lines) compared to the dopamine-free baseline (black line). (f–h) Dependence of (f) SIDP, (g) SNDP, and (h) SFDP of EPSCs on dopamine concentration.

In biological nervous systems, the concentration of neurotransmitters (e.g., dopamine) within the synaptic cleft is a critical determinant of signal transmission efficiency. This concentration-dependent modulation was faithfully recapitulated in our device. By introducing varying dopamine concentrations (2 μM, 20 μM, and 200 μM) into the electrolyte, we observed a progressive attenuation of both the IPSC (Figure 4d, bottom) and EPSC (Figure 5c, bottom) amplitudes, as well as their associated memory effects. Quantitatively, the maximum changes in IPSC or EPSC after receiving gate voltage pulses (defined as ΔIPSC_max_ or ΔEPSC_max_) exhibited a distinct negative correlation with dopamine concentration. This trend effectively mimics the biological mechanism whereby elevated dopamine levels induce stronger suppression of postsynaptic activity.

Furthermore, the long-term stability of the synaptic weight modulation was quantitatively evaluated by extracting the retention times from the postpulse relaxation curves (Figure 4e). The retention time—defined as the characteristic duration for maintaining the modulated synaptic weight—exhibited a concentration-dependent decrease from approximately 3.9 × 10^3^ s in PBS to 1.7 × 10^3^ s in the presence of 200 μM dopamine. Despite this decrease, the retention times consistently exceeded 1000 s across all tested concentrations, confirming that the dopamine-induced inhibition constitutes a genuine LTP behavior rather than a transient fluctuation.

Beyond the static attenuation of signal amplitude, the temporal dynamics of synaptic transmission are equally critical for information processing. Therefore, we first evaluated paired-pulse depression (PPD) and paired-pulse facilitation (PPF), phenomena in which the response to an initial presynaptic signal is decreased or increased by a subsequent one, to characterize the device’s temporal recognition capabilities. The PPD and PPF indices are defined as the ratio of the second pulse response to the first (Δ*G*_2_/Δ*G*_1_, inset of Figure 4f for PPD index, and inset of Figure 5d for PPF index). To demonstrate PPD, two identical positive pulses (*V*_G_ = +0.5 V, *t* = 2s) with varying intervals (Δ*t*, 0.5 s–100 s) were applied. In the absence of dopamine, the maximum PPD index reached ∼132% at Δ*t* = 0.5 s (Figure 4f, black line). However, increasing dopamine concentration caused a distinct decline in PPD index: for instance, 200 μM dopamine reduced the maximum PPD index to ∼121% (Figure 4f, green line). Crucially, at a fixed dopamine concentration, the PPD index decayed monotonically as a function of Δ*t*, in good agreement with the behavior of biological synapses. Similarly, PPF behavior was demonstrated using paired negative pulses (Figure 5d, *V*_G_ = −0.5 V). The PPF index also declined with increasing Δ*t*, and higher dopamine concentrations (0 to 20 μM) suppressed the PPF index from ∼119% to ∼112% (at Δ*t* = 0.5 s). Building on these plasticity results, we emulated physiological learning and forgetting processes by applying alternating negative and positive pulses (Figure 5e). In the absence of dopamine, the channel current readily exceeded the thresholds for learning (Threshold 1) and forgetting (Threshold 2). In contrast, dopamine administration simultaneously attenuated both EPSCs and IPSCs, thereby preventing the currents from reaching these thresholds. This demonstrates an inhibitory modulation of the learning and forgetting capabilities, simulating the gain control function in biological systems.

Accompanying these sophisticated synaptic functions, the operational robustness and chemical reversibility of the device are paramount for sustained neuromorphic performance. To evaluate the device’s stability under high-concentration conditions, we first monitored the ΔIPSC response over 1000 consecutive positive pulses (*V*_G_ = +0.5 V, *t* = 2s) in the presence of 200 μM dopamine (Figure S5). The modulation magnitude remained remarkably consistent over the 4000 s test period, indicating that the electrochemical oxidation of dopamine does not lead to significant electrode fouling or the formation of an insulating polydopamine layer within this time frame. In parallel, the cyclic stability of the physiological learning and forgetting processes (Figure 5e) was verified over 100 continuous cycles (Figure S6). The device exhibited reproducible current modulation for over 16,000 s, confirming its robust performance during prolonged operation. Notably, this dopamine-induced attenuation is highly reversible: upon flushing the secondary electrolyte with fresh PBS, both IPSC and EPSC amplitudes significantly recovered to their near-original values (Figure S7). This recovery confirms that the observed inhibition stems from a reversible electrochemical dedoping process rather than irreversible material degradation.

Furthermore, we confirmed that the inhibitory effect of dopamine remains effective under various presynaptic pulse conditions. By monitoring IPSCs and EPSCs while varying pulse amplitude, number, and duration (Figures 4g–i and 5f–h), we observed that the fundamental correlations between synaptic weight and pulse energy were preserved, although their magnitudes were reduced in the presence of dopamine. As expected, at any given dopamine concentration, ΔIPSC_max_ showed a positive correlation with positive pulse amplitude (Figure 4g), pulse number (Figure 4h), and duration (Figure 4i). Similarly, ΔEPSC_max_ exhibited a positive correlation with negative pulse amplitude (Figure 5f), pulse number (Figure 5g), and duration (Figure 5h), confirming that the device’s fundamental switching mechanism remains intact. Meanwhile, superimposed on these trends, a strong negative correlation with dopamine concentration was observed under all identical pulse conditions. Collectively, these results demonstrate that the device effectively mimics biological SIDP, SNDP, and SFDP behaviors, while successfully integrating the overarching attenuation control mediated by neurotransmitters.

To pinpoint the origin of the dopamine sensitivity in our device, we investigated the synaptic behavior of a control device lacking the PEDOT:PSS thin film on its floating gate (Figure S8, top). Under identical gate voltage pulses, in contrast to the standard device, the control device exhibited negligible changes in synaptic behavior upon dopamine introduction (Figure S8, bottom). This result definitively confirms that the PEDOT:PSS film on the floating gate is essential for mediating neurotransmitter-induced signal attenuation.

Based on these findings, we propose a mechanism underlying the observed dopamine-induced attenuative process. The floating gate structure, together with the ion gel forming the primary electrolyte, collectively governs the synaptic behavior of our device, including both STP and LTP. Specifically, when a positive pulse (*V*_G_) is applied to the gate electrode (Figure 6a, black line, bottom-left), it is distributed across two series components: the PEDOT:PSS portion of the floating gate and the ion gel. This relationship is described by

1 $$V_{G}=V_{PEDOT}+V_{FG}$$

where *V*_PEDOT_ is the voltage drop across the floating gate’s PEDOT:PSS portion, and *V*_FG_ represents the potential drop across the ion gel (equivalent to the floating gate potential relative to ground).

**Figure 6:**
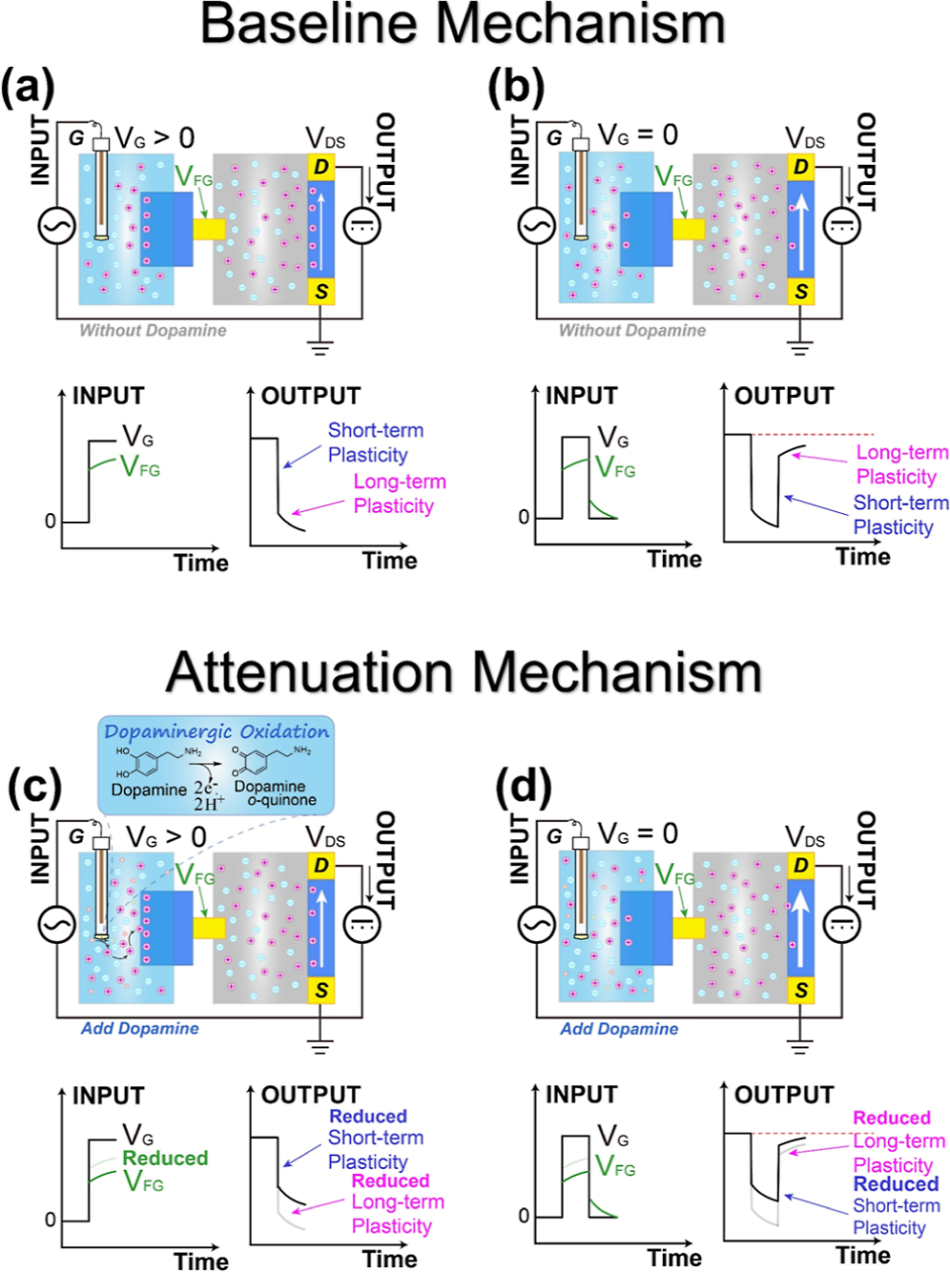
Schematic illustration of the operating principle of the artificial synapse. (a,b) Baseline mechanism: in the absence of dopamine, the gate pulse drives ion injection and migration in the ion gel, establishing STP and LTP (a), followed by ionic relaxation upon pulse removal (b). (c,d) Attenuation mechanism: in the presence of dopamine, proton generation via oxidation leads to further dedoping of the floating gate PEDOT:PSS. This increases the gate circuit resistance (*R*_PEDOT_) and lowers the effective potential across the ion gel (*V*_FG_) (c), resulting in attenuated STP and LTP amplitudes during both the stimulation and relaxation phases (d).

In the absence of dopamine (baseline mechanism, Figures 6a,b), applying a positive pulse induces a change in *V*_FG_ (Figure 6a, green line, bottom-left), which can be directly observed by connecting an oscilloscope probe to the gold electrode of the floating gate (Figure S10a, black line, bottom). Notably, *V*_FG_ does not instantaneously track *V*_G_ due to the kinetic constraints of ion migration in the PBS and injection into the PEDOT:PSS. In the primary electrolyte (ion gel) of our device, the changing *V*_FG_ directly modulates the motion of cations and anions. As a result, cations near the channel surface are driven from the electrolyte into the PEDOT:PSS channel (Figure 6a, top), resulting in channel dedoping and thereby generating the STP of IPSC (Figure 6a, bottom-right). At the same time, the bulky ions in the ion gel prevent the ion injection process from being accomplished instantaneously. Instead, the remaining cations continue to flow and migrate slowly as *V*_G_ and *V*_FG_ are maintained, contributing to the LTP of IPSC (Figure 6a, bottom-right). A similar process occurs when *V*_G_ returns to its original value (Figure 6b). The sudden drop of *V*_FG_ generates STP, whereas the gradual migration of bulky ions contributes to LTP.

Meanwhile, in the presence of dopamine (attenuation mechanism, Figures 6c,d), the above voltage distribution is fundamentally altered by dopamine oxidation, leading to attenuation of both STP and LTP of the IPSC. Specifically, when the same positive gate pulse is applied (Figure 6c, black line, bottom-left), the electrochemical oxidation of dopamine is simultaneously triggered and accelerated, generating additional electrons and protons (cations) in the secondary aqueous electrolyte (Figure 6c, top). Concurrently, the electric field induced by the gate voltage drives electrons toward the external circuit via the control gate electrode, while the additional protons migrate through the aqueous electrolyte toward the floating gate electrode. Since only the PEDOT:PSS portion of the floating gate is in contact with the aqueous electrolyte, these additional protons are injected into the floating gate PEDOT:PSS, causing its further dedoping and, crucially, an increase in its electrical resistance (*R*_PEDOT_). Notably, since the ion gel is physically isolated from this reaction in the aqueous electrolyte, its impedance remains unchanged. According to the voltage divider principle (eq 1), when the *V*_G_ remains constant and the ion gel is essentially unaffected, a further increase in *R*_PEDOT_ causes a larger proportion of *V*_G_ to drop across the PEDOT:PSS layer, resulting in an increase in *V*_PEDOT_. Consequently, the effective potential applied to the ion gel (*V*_FG_) is significantly reduced (Figure 6c, green line, bottom-left), with this effect becoming more pronounced at higher dopamine concentrations (Figure S10a, other lines, bottom). This reduced driving force (*V*_FG_) limits cation injection into the channel, thereby attenuating both the STP and LTP of the IPSC (Figure 6c, bottom-right). When *V*_G_ returns to its original value (Figure 6d), the *V*_FG_ reverts to a value which is lower than that without dopamine (Figure 6d, green line, bottom-left). Combined with the fact that the ΔIPSC previously induced by the gate voltage has already been weakened by dopamine oxidation, this results in STP and LTP that are reduced compared to those observed without dopamine (Figure 6d, bottom-right).

To further validate the proposed mechanism, the resistance of the floating-gate PEDOT:PSS film was directly monitored during dopamine exposure (see Experimental Section/Experimental and Figure S11 for the experimental setup). The results reveal a clear concentration-dependent increase in resistance, which we attribute to the chemical dedoping of PEDOT:PSS during the catalytic oxidation of dopamine (Figure S12). This direct measurement confirms that the attenuation of synaptic weight is governed by the modulation of gate impedance rather than channel conductance changes resulting from ion implantation alone.

It is worth noting that the dopamine-induced attenuation mechanism is not limited to positive pulses, but also affects EPSCs induced by negative gate pulses (Figure S10b). Specifically, the oxidation of dopamine similarly suppresses the *V*_FG_ modulation during negative voltage applications, contributing to the attenuation of ΔEPSC. However, we observed a distinct asymmetry in the magnitude of these responses: the ΔEPSC induced by negative pulses is notably smaller than the ΔIPSC induced by positive pulses. This discrepancy is attributed to the steric difference between the ionic species in the primary ion gel electrolyte. The anions, which are driven into the channel under negative gate bias to induce EPSCs, possess a larger ionic radius and lower mobility compared to the cations responsible for IPSCs. Consequently, the migration and injection of anions are kinetically more restricted, resulting in a less pronounced modulation of the channel conductivity compared to the cation-dominated process.

While our OECT-based synapse successfully emulates dopamine-induced attenuation, chemical selectivity remains a concern for real-time biological use. Common electroactive interferents (e.g., ascorbic acid and uric acid) have similar oxidation potentials and may affect the gate response. Our current study leverages dopamine’s characteristic redox peak to achieve localized detection. For future biointegrated applications, charge-selective coatings^64^ or catalytic modifiers^53^ on the gate electrode can help isolate the target neurotransmitter signal from the background biochemical noise.

## Conclusion

In summary, we demonstrate an OECT-based artificial synapse capable of chemical communication, in which both inhibitory and excitatory synaptic behaviors exhibit dopamine-induced attenuation, mirroring the behavior of glutamatergic and GABAergic synapses in the biological nucleus accumbens. The device exhibits typical IPSC and EPSC characteristics, including STP and LTP, memory effects under gate voltage pulses, and PPD/PPF behavior under paired pulses, closely resembling the inhibitory and excitatory biological synapses. Upon adding dopamine to the secondary aqueous electrolyte, the aforementioned synaptic behaviors are weakened, likely due to dopamine oxidation, which induces further dedoping of PEDOT:PSS in the floating gate and a resulting decrease in floating gate voltage (*V*_FG_). These features mimic the biological phenomenon in which dopamine attenuates certain inhibitory and excitatory synaptic signals. Our strategy provides a facile approach to chemically modulate artificial synapses in an inhibitory manner, opening new avenues for the development of artificial synaptic devices, which are key components for constructing neuromorphic computing systems, neuro-prosthetics, and human–machine interfaces.

## Materials and Methods

### Materials

Poly­(3,4-ethylenedioxythiophene):poly­(styrenesulfonate) (PEDOT:PSS) aqueous solution and phosphate buffered saline (PBS) solution were purchased from Sigma-Aldrich and stored at 4 °C. 1-Ethyl-3-methylimidazolium bis­(trifluoromethylsulfonyl) imide ([EMIM]­[TFSI]) and poly­(vinylidene fluoride-*co*-hexafluoropropylene) (P­(VDF-HFP)) were obtained from Sigma-Aldrich Co., Ltd. All other reagents used were purchased from Tokyo Chemical Industry Co., Ltd.

The modified PEDOT:PSS aqueous solution was prepared by adding 6 vol % ethylene glycol to increase the PEDOT:PSS conductivity, 0.1 vol % dodecylbenzenesulfonic acid as a surfactant, and 1 vol % (3-glycidyloxypropyl)­trimethoxysilane as a cross-linking agent to improve mechanical stability.

To fabricate the solid ion gel films, 0.1 g of P­(VDF-HFP) was dispersed into 0.7 g of acetone and stirred for 3 h on a hot plate at 60 °C. Subsequently, 0.4 g of [EMIM]­[TFSI] was added to the aforementioned solution, and the mixture was stirred for 10 min at room temperature. Right after that, this mixed solution was drop-cast on a glass slide, then left at room temperature for more than 48 h, resulting in the formation of the ion gel film for future use.

The polydimethylsiloxane (PDMS) well was prepared by mixing PDMS precursor with a cross-linker at a ratio of 20:1 wt/wt and degassed in a vacuum desiccator before being poured onto the mold to form holes, and then heated to 80 °C for 2 h.

### Preparation of Floating Gate OECT-Based Artificial Synaptic Device

First, patterned Cr/Au source, drain, and gate electrodes were deposited on the surface of glass substrates through a shadow mask by thermal evaporation (SANYU Electron SVC-700TM) (Figure S1a). A thin layer of Cr (thickness: ∼5 nm) was used as an adhesion layer for the Au layer (thickness: ∼40 nm).

Subsequently, after the cleaned glass substrate was exposed to O_2_ plasma for 5 min (Harrick PDC-32G-2, 18 W), two PEDOT:PSS layers (thickness: ∼80 nm) were prepared by spin-coating the modified PEDOT:PSS aqueous solution between the source and drain electrodes as the channel and onto the floating-gate electrode at 2000 rpm for 1 min, immediately followed by thermal annealing at 120 °C for 30 min in the air.

After cooling down, the prepared solid ion gel film (primary electrode) was put onto the glass substrate, covering the PEDOT:PSS channel and a part of the Cr/Au floating-gate electrode. After the film was formed, a PDMS well was attached to the glass substrate around the PEDOT:PSS floating-gate electrode to immobilize the secondary aqueous electrolyte (PBS solution).

### Electrical Measurements

For the electrical measurements, a silver/silver chloride (Ag/AgCl) standard electrode is directly inserted into the secondary aqueous electrolyte, acting as the control gate electrode.

A Keithley 4200A-SCS parameter analyzer was used to control the voltages of gate, source, and drain electrodes and record the currents through the channel (channel current, *I*_DS_).

For the measurements of transfer curves, a constant DC voltage was applied onto the drain electrode (*V*_DS_ = 0.1 V) and the source electrode was grounded, while the gate voltage swept from 0 to +1 V. The change in the channel current (*I*_DS_) was also recorded to graph the transfer curves. To graph the transfer curves of the device at different dopamine concentrations, this process was repeated after dopamine additions (into the PDMS well) with increasing concentrations.

For the real time measurements, a constant DC voltage was applied to the gate electrode (*V*_G_ = 0.3 V) and the drain electrode (*V*_DS_ = 0.1 V), respectively, while the source electrode was grounded. Subsequently, dopamine solution was added to the device, and the channel current (*I*_DS_) was monitored and recorded.

For the measurements of synaptic behaviors of the device under voltage pulses, a constant DC voltage was applied to the drain electrode (*V*_DS_ = 0.1 V), and the source electrode was grounded, while programmed voltage pulses were applied onto the gate electrode (for instance, a voltage pulse with an amplitude of 0.5 V and a duration of 2 s). To record the synaptic behaviors of the device at different dopamine concentrations, this process was repeated after dopamine additions with increasing concentrations.

For the measurements of the voltages of the Cr/Au floating gate electrode (*V*_FG_), a probe connected with an oscilloscope (Tektronix TDS 2012B, the other end grounded) was attached to the Cr/Au electrode (Figure S9), recording the changes in *V*_FG_ during the measurements of synaptic behaviors of the device. For the measurements of the floating-gate PEDOT:PSS resistance, another Cr/Au electrode was deposited on the surface of glass substrates next to the Cr/Au floating-gate electrode, both of which are connected by the floating-gate PEDOT:PSS (Figure S11). By applying a small constant measurement voltage (*V*_Meas_ = 10 mV) across the PEDOT:PSS film between the new probing electrode and the Cr/Au floating gate, the floating-gate PEDOT:PSS resistance can be recorded. After implementing different concentration of dopamine in the aqueous electrolyte, we record the resistance at the last point of the same gate voltage pulse (+0.5 V, 2 s) in order to compare the effect from the oxidation of dopamine.

## Supplementary Material



## Notes

Additional experimental details, including working mechanism of OECT-based biosensors; schematic of the devices and electrical measurements; characterizations of PEDOT:PSS films; transconductance of the device; IPSCs and EPSCs, along with the cumulative memory effect of the device under 5 successive positive or negative gate voltage pulses; real-time response of the device to the addition of different concentrations of dopamine; long-term reliability and stability tests; synaptic behaviors of the control device; schematic of the probe and the oscilloscope; floating gate voltage versus gate voltage with and without dopamine in the device; schematic of detecting resistance changes of the floating-gate PEDOT:PSS at different dopamine concentrations (PDF)

## Abbreviations

OECT: organic electrochemical transistor
ACh: acetylcholine
GABA: γ-aminobutyric acid
STP: short-term plasticity
LTP: long-term plasticity
PPF: paired-pulse facilitation
PPD: paired-pulse depression
EPSC: excitatory postsynaptic current
IPSC: inhibitory postsynaptic current
PEDOT:PSS: poly­(3,4-ethylenedioxythiophene):poly­(styrenesulfonate)
P­(VDF-HFP): poly­(vinylidene fluoride-*co*-hexafluoropropylene)
SEM: scanning electron microscopy
FTIR: Fourier-transform infrared spectroscopy
SIDP: spike intensity-dependent plasticity
SNDP: spike number-dependent plasticity
SFDP: spike frequency-dependent plasticity
[EMIM]­[TFSI]: 1-ethyl-3-methylimidazolium bis­(trifluoromethylsulfonyl) imide
PDMS: polydimethylsiloxane

## References

[1] Christensen D. V., Dittmann R., Linares-Barranco B., Sebastian A., Le Gallo M., Redaelli A., Slesazeck S., Mikolajick T., Spiga S., Menzel S. (2022). roadmap
on neuromorphic computing and engineering. Neuromorph.
Comput. Eng..

[2] Akarvardar K., Wong H.-S. P. (2023). Technology prospects
for data-intensive computing. Proc. IEEE.

[3] Wang Y., Chen S., Cheng X., Chen W., Xiong Z., Lv Z., Wu C., Wang L., Zhang G., Zhu X. (2024). Neurotransmitter-Mediated
Plasticity in 2D Perovskite Memristor for
Reinforcement Learning. Adv. Funct. Mater..

[4] Backus J. (1978). Can programming
be liberated from the von Neumann style? a functional style and its
algebra of programs. Commun. ACM.

[5] Chen F., Zhou Y., Zhu Y., Zhu R., Guan P., Fan J., Zhou L., Valanoor N., Von Wegner F., Saribatir E. (2021). Recent progress in artificial
synaptic devices:
materials, processing and applications. J. Mater.
Chem. C.

[6] Lu K., Li X., Sun Q., Pang X., Chen J., Minari T., Liu X., Song Y. (2021). Solution-processed electronics for artificial synapses. Mater. Horiz..

[7] van
de Burgt Y., Lubberman E., Fuller E. J., Keene S. T., Faria G. C., Agarwal S., Marinella M. J., Alec Talin A., Salleo A. (2017). A non-volatile organic electrochemical
device as a low-voltage artificial synapse for neuromorphic computing. Nat. Mater..

[8] Roy K., Jaiswal A., Panda P. (2019). Towards spike-based machine intelligence
with neuromorphic computing. Nature.

[9] Zhang W., Gao B., Tang J., Yao P., Yu S., Chang M.-F., Yoo H.-J., Qian H., Wu H. (2020). Neuro-inspired computing
chips. Nat. Electron..

[10] Furber S. (2016). Large-scale
neuromorphic computing systems. J. Neural Eng..

[11] Wan C., Cai P., Wang M., Qian Y., Huang W., Chen X. (2020). Artificial
Sensory Memory. Adv. Mater..

[12] Cheng M., Xie Y., Wang J., Jin Q., Tian Y., Liu C., Chu J., Li M., Li L. (2025). Neurotransmitter-mediated artificial
synapses based on organic electrochemical transistors for future biomimic
and bioinspired neuromorphic systems. J. Semicond..

[13] Zeilhofer H. U., Wildner H., Yévenes G. E. (2012). Fast synaptic
inhibition in spinal
sensory processing and pain control. Physiol.
Rev..

[14] Starr M. S. (1996). The role
of dopamine in epilepsy. Synapse.

[15] Pereda A. E. (2014). Electrical
synapses and their functional interactions with chemical synapses. Nat. Rev. Neurosci..

[16] Sabandal J. M., Berry J. A., Davis R. L. (2021). Dopamine-based mechanism for transient
forgetting. Nature.

[17] Speranza L., di Porzio U., Viggiano D., de Donato A., Volpicelli F. (2021). Dopamine:
The Neuromodulator of Long-Term Synaptic
Plasticity, Reward and Movement Control. Cells.

[18] Fuchsberger T., Stockwell I., Woods M., Brzosko Z., Greger I. H., Paulsen O. (2025). Dopamine increases protein synthesis
in hippocampal
neurons, enabling dopamine-dependent LTP. eLife.

[19] Nicola S. M., Malenka R. C. (1997). Dopamine depresses excitatory and inhibitory synaptic
transmission by distinct mechanisms in the nucleus accumbens. J. Neurosci..

[20] Uchida N. (2014). Bilingual
neurons release glutamate and GABA. Nat. Neurosci..

[21] Pereda A. E., Curti S., Hoge G., Cachope R., Flores C. E., Rash J. E. (2013). Gap junction-mediated
electrical transmission: Regulatory
mechanisms and plasticity. Biochim. Biophys.
Acta, Biomembr..

[22] Calabresi P., Picconi B., Tozzi A., Di Filippo M. (2007). Dopamine-mediated
regulation of corticostriatal synaptic plasticity. Trends Neurosci..

[23] Shim H., Ershad F., Patel S., Zhang Y., Wang B., Chen Z., Marks T. J., Facchetti A., Yu C. (2022). An elastic and reconfigurable synaptic transistor based on a stretchable
bilayer semiconductor. Nat. Electron..

[24] Qin W., Kang B. H., Kim H. J. (2021). Flexible
artificial synapses with
a biocompatible Maltose–Ascorbic acid electrolyte gate for
neuromorphic computing. ACS Appl. Mater. Interfaces.

[25] Zhang Y., Ye G., van der
Pol T. P., Dong J., van Doremaele E. R., Krauhausen I., Liu Y., Gkoupidenis P., Portale G., Song J. (2022). High-performance organic
electrochemical transistors and neuromorphic devices comprising naphthalenediimide-dialkoxybithiazole
copolymers bearing glycol ether pendant groups. Adv. Funct. Mater..

[26] He K., Liu Y., Wang M., Chen G., Jiang Y., Yu J., Wan C., Qi D., Xiao M., Leow W. R. (2020). An
artificial somatic reflex arc. Adv. Mater..

[27] Zang Y., Shen H., Huang D., Di C. A., Zhu D. (2017). A dual-organic-transistor-based
tactile-perception system with signal-processing functionality. Adv. Mater..

[28] Shim H., Sim K., Ershad F., Yang P., Thukral A., Rao Z., Kim H.-J., Liu Y., Wang X., Gu G., Gao L., Wang X., Chai Y., Yu C. (2019). Stretchable elastic
synaptic transistors for neurologically integrated soft engineering
systems. Sci. Adv..

[29] Wan C., Cai P., Guo X., Wang M., Matsuhisa N., Yang L., Lv Z., Luo Y., Loh X. J., Chen X. (2020). An artificial sensory neuron with visual-haptic fusion. Nat. Commun..

[30] Liu X., Wang D., Chen W., Kang Y., Fang S., Luo Y., Luo D., Yu H., Zhang H., Liang K. (2024). Optoelectronic synapses
with chemical-electric behaviors in gallium
nitride semiconductors for biorealistic neuromorphic functionality. Nat. Commun..

[31] Lee Y., Oh J. Y., Xu W., Kim O., Kim T. R., Kang J., Kim Y., Son D., Tok J. B.-H., Park M. J., Bao Z., Lee T. W. (2018). Stretchable
organic
optoelectronic sensorimotor synapse. Sci. Adv..

[32] Yan X., Zheng Z., Sangwan V. K., Qian J. H., Wang X., Liu S. E., Watanabe K., Taniguchi T., Xu S.-Y., Jarillo-Herrero P. (2023). Moiré synaptic
transistor with room-temperature neuromorphic functionality. Nature.

[33] Wei H., Shi R., Sun L., Yu H., Gong J., Liu C., Xu Z., Ni Y., Xu J., Xu W. (2021). Mimicking
efferent
nerves using a graphdiyne-based artificial synapse with multiple ion
diffusion dynamics. Nat. Commun..

[34] Qian C., Sun J., Kong L.-a., Gou G., Yang J., He J., Gao Y., Wan Q. (2016). Artificial
Synapses Based on in-Plane Gate Organic
Electrochemical Transistors. ACS Appl. Mater.
Interfaces.

[35] Melianas A., Quill T. J., LeCroy G., Tuchman Y., Loo H. v., Keene S. T., Giovannitti A., Lee H. R., Maria I. P., McCulloch I., Salleo A. (2020). Temperature-resilient solid-state
organic artificial synapses for neuromorphic computing. Sci. Adv..

[36] Wang Y., Zhang Q., Astier H. P., Nickle C., Soni S., Alami F. A., Borrini A., Zhang Z., Honnigfort C., Braunschweig B. (2022). Dynamic molecular switches with hysteretic
negative differential conductance emulating synaptic behaviour. Nat. Mater..

[37] Kim K.-N., Sung M.-J., Park H.-L., Lee T.-W. (2022). Organic
Synaptic
Transistors for Bio-Hybrid Neuromorphic Electronics. Adv. Electron. Mater..

[38] Ling H., Koutsouras D. A., Kazemzadeh S., Van De Burgt Y., Yan F., Gkoupidenis P. (2020). Electrolyte-gated transistors for synaptic electronics,
neuromorphic computing, and adaptable biointerfacing. Appl. Phys. Rev..

[39] Torricelli F., Adrahtas D. Z., Bao Z., Berggren M., Biscarini F., Bonfiglio A., Bortolotti C. A., Frisbie C. D., Macchia E., Malliaras G. G. (2021). Electrolyte-gated transistors for enhanced
performance bioelectronics. Nat. Rev. Methods
Primers.

[40] Yu T.-F., Chen H.-Y., Liao M.-Y., Tien H.-C., Chang T.-T., Chueh C.-C., Lee W.-Y. (2020). Solution-Processable
Anion-doped
Conjugated Polymer for Nonvolatile Organic Transistor Memory with
Synaptic Behaviors. ACS Appl. Mater. Interfaces.

[41] Yan Y., Chen Q., Wu X., Wang X., Li E., Ke Y., Liu Y., Chen H., Guo T. (2020). High-performance organic
electrochemical transistors with nanoscale channel length and their
application to artificial synapse. ACS Appl.
Mater. Interfaces.

[42] Gerasimov J. Y., Gabrielsson R., Forchheimer R., Stavrinidou E., Simon D. T., Berggren M., Fabiano S. (2019). An evolvable organic
electrochemical transistor for neuromorphic applications. Adv. Sci..

[43] Lee S. K., Cho Y. W., Lee J. S., Jung Y. R., Oh S. H., Sun J. Y., Kim S., Joo Y. C. (2021). Nanofiber channel
organic electrochemical transistors for low-power neuromorphic computing
and wide-bandwidth sensing platforms. Adv. Sci..

[44] Gkoupidenis P., Schaefer N., Strakosas X., Fairfield J. A., Malliaras G. G. (2015). Synaptic plasticity functions in
an organic electrochemical
transistor. Appl. Phys. Lett..

[45] Hu J., Jing M.-J., Huang Y.-T., Kou B.-H., Li Z., Xu Y.-T., Yu S.-Y., Zeng X., Jiang J., Lin P., Zhao W.-W. (2024). A Photoelectrochemical Retinomorphic Synapse. Adv. Mater..

[46] Yang Y., Wu Y., He W., Tien H., Yang W., Michinobu T., Chen W., Lee W., Chueh C. (2022). Tuning Ambipolarity
of the Conjugated Polymer Channel Layers of Floating-Gate Free Transistors:
From Volatile Memories to Artificial Synapses. Adv. Sci..

[47] Li Z., Liu F.-Q., Wu Q.-Q., Chen M.-H., Zhu Y.-C., Zhao W.-W. (2025). Neuromorphic Phototransistor
with Biochemical Reconfigurability. ACS Nano.

[48] Chen Q.-G., Liao W.-T., Li R.-Y., Sanjuán I., Hsiao N.-C., Ng C.-T., Chang T.-T., Guerrero A., Chueh C.-C., Lee W.-Y. (2025). Organic Solid-State
Electrolyte Synaptic
Transistors with Photoinduced Thiol–Ene Cross-linked Polymer
Electrolytes for Deep Neural Networks. ACS Mater.
Lett..

[49] Liu C.-J., Hsiao S.-W., Chen Q.-G., Hong Q.-A., Lin Y.-T., Chueh C.-C., Ng C.-T., Chang T.-T., Kim S. H., Chiu Y.-C., Lee W.-Y. (2026). Polarizable
Thiol–Ene Cross-Linked
Nitrile Dielectrics for Stretchable Low-Voltage Neuromorphic Transistors
with Acoustic Classification. ACS Appl. Mater.
Interfaces.

[50] Keene S. T., Lubrano C., Kazemzadeh S., Melianas A., Tuchman Y., Polino G., Scognamiglio P., Cinà L., Salleo A., van de Burgt Y. (2020). A biohybrid synapse
with neurotransmitter-mediated plasticity. Nat.
Mater..

[51] Wang T., Wang M., Wang J., Yang L., Ren X., Song G., Chen S., Yuan Y., Liu R., Pan L., Li Z., Leow W. R., Luo Y., Ji S., Cui Z., He K., Zhang F., Lv F., Tian Y., Cai K., Yang B., Niu J., Zou H., Liu S., Xu G., Fan X., Hu B., Loh X., Wang L., Chen X. (2022). A chemically mediated artificial neuron. Nat.
Electron..

[52] Shao L., Luo S., Wang Z., Xu X., Yan Y., Wu Y., Guo M., Wei D., Zhao Y., Liu Y. (2022). A flexible biohybrid
reflex arc mimicking neurotransmitter transmission. Cell Rep. Phys. Sci..

[53] Xu X., Zhang H., Shao L., Ma R., Guo M., Liu Y., Zhao Y. (2023). An Aqueous Electrolyte Gated Artificial Synapse with
Synaptic Plasticity Selectively Mediated by Biomolecules. Angew. Chem., Int. Ed..

[54] Kim J., Campbell A. S., de Ávila B. E.-F., Wang J. (2019). Wearable biosensors
for healthcare monitoring. Nat. Biotechnol..

[55] Kim D., Lee J. S. (2022). Neurotransmitter-Induced
Excitatory and Inhibitory
Functions in Artificial Synapses. Adv. Funct.
Mater..

[56] Lubrano C., Bruno U., Ausilio C., Santoro F. (2022). Supported Lipid Bilayers
Coupled to Organic Neuromorphic Devices Modulate Short-Term Plasticity
in Biomimetic Synapses. Adv. Mater..

[57] He K., Wang C., He Y., Su J., Chen X. (2023). Artificial
Neuron Devices. Chem. Rev..

[58] Xu Q., Chen J., Li Y., Qiu J., Liu X., Cao J., Chen Y., Liu M., Wang M. (2024). Fully Printed Dual-Gate
Organic Electrochemical Synaptic Transistor With Neurotransmitter-Mediated
Plasticity. IEEE Electron Device Lett..

[59] Yang A., Li Y., Yang C., Fu Y., Wang N., Li L., Yan F. (2018). Fabric organic electrochemical
transistors for biosensors. Adv. Mater..

[60] Lasater E. M., Dowling J. E. (1985). Dopamine decreases conductance of
the electrical junctions
between cultured retinal horizontal cells. Proc.
Natl. Acad. Sci. U.S.A..

[61] White S. P., Dorfman K. D., Frisbie C. D. (2016). Operating and sensing mechanism of
electrolyte-gated transistors with floating gates: Building a platform
for amplified biodetection. J. Phys. Chem. C.

[62] Chang T., Jo S.-H., Lu W. (2011). Short-term
memory to long-term memory
transition in a nanoscale memristor. ACS Nano.

[63] Ben-Ari Y. (2002). Excitatory
actions of GABA during development: the nature of the nurture. Nat. Rev. Neurosci..

[64] Ali M., Khalid M. A. U., Kim Y. S., Soomro A. M., Hussain S., Doh Y. H., Choi K. H. (2021). MWCNTs/PEDOT:PSS Composite as Guiding
Layer on Screen-Printed Carbon Electrode for Linear Range Lactate
Detection. J. Electrochem. Soc..

