# Supplementary material for: Dopamine-Mediated Attenuation of OECT-Based Aqueous Artificial Chemical Synapses
Source: ACS Appl Mater Interfaces. 2026 May 5;18(19):27888–98. doi: 10.1021/acsami.6c02711 (PMC13195573; doi:10.1021/acsami.6c02711)
Supplement: Supplementary file 1 [file am6c02711_si_001.pdf]

Supporting Information

Dopamine-Mediated Attenuation of OECT-Based Aqueous Artificial Chemical Synapses

Haoqin Zhang,[1] Xinzhao Xu[2], Waner He[1], Atsushi Isobe[1], Yunqi Liu[,2], Yan Zhao[*,2] and Tsuyoshi Michinobu*[,1]*

[1] Department of Materials Science and Engineering, Institute of Science Tokyo, 2-12-1 Ookayama, Meguro-ku, Tokyo 152-8552, Japan.

E-mail: michinobu.t.aa@m.titech.ac.jp (T.M.)

[2] Department of Materials Science, Fudan University, Shanghai 200433, P. R. China.

E-mail: liuyq@fudan.edu.cn (Y.L.); zhaoy@fudan.edu.cn (Y.Z.)

1. Working mechanism of OECT-based biosensors:

The working mechanism of OECT-based biosensors, the physics underlying OECT-based sensors and the connection between the response of the device and the analyte concentration in OECT-based enzymatic sensors have been detailed in previous publication.[1,2]

Briefly, the working mechanism of our device is governed by the device physics of floating-gate OECTs and electrochemical redox kinetics. As shown in the potential diagram (based on the Bernards model), an electric field is established between the gate electrode and the floating-gate electrode when the device is in operation. This electric field drives the cations (including the protons generated from the oxidation of dopamine) in the electrolyte to flow and eventually penetrate into the organic layer.

Since ions can be injected into the entire volume of the floating-gate PEDOT:PSS film (i.e., volumetric capacitance), they change the doping state of the organic film and modulate the conductance of the floating-gate PEDOT:PSS. The redox reaction of dopamine on the gate electrode induces a potential change according to the Nernst equation. Since the gate potential of the OECT is fixed (V_G is constant), the redox reaction causes a decrease in the primary electrolyte (ion gel) relative to that of the gate. Therefore, the addition of dopamine is equivalent to the situation where a smaller effective voltage (V_{eff}) is applied to the gate.

Based on the Bernards model and the Nernst equation, V_{eff} is logarithmically dependent on the concentration of dopamine:

$$V_{eff} = \beta \log[Dopamine] + \gamma \quad (1)$$

where β and γ are constants. Consequently, the channel current (synaptic weight) is sensitive to the concentration of dopamine in the electrolyte.

Supporting Figures

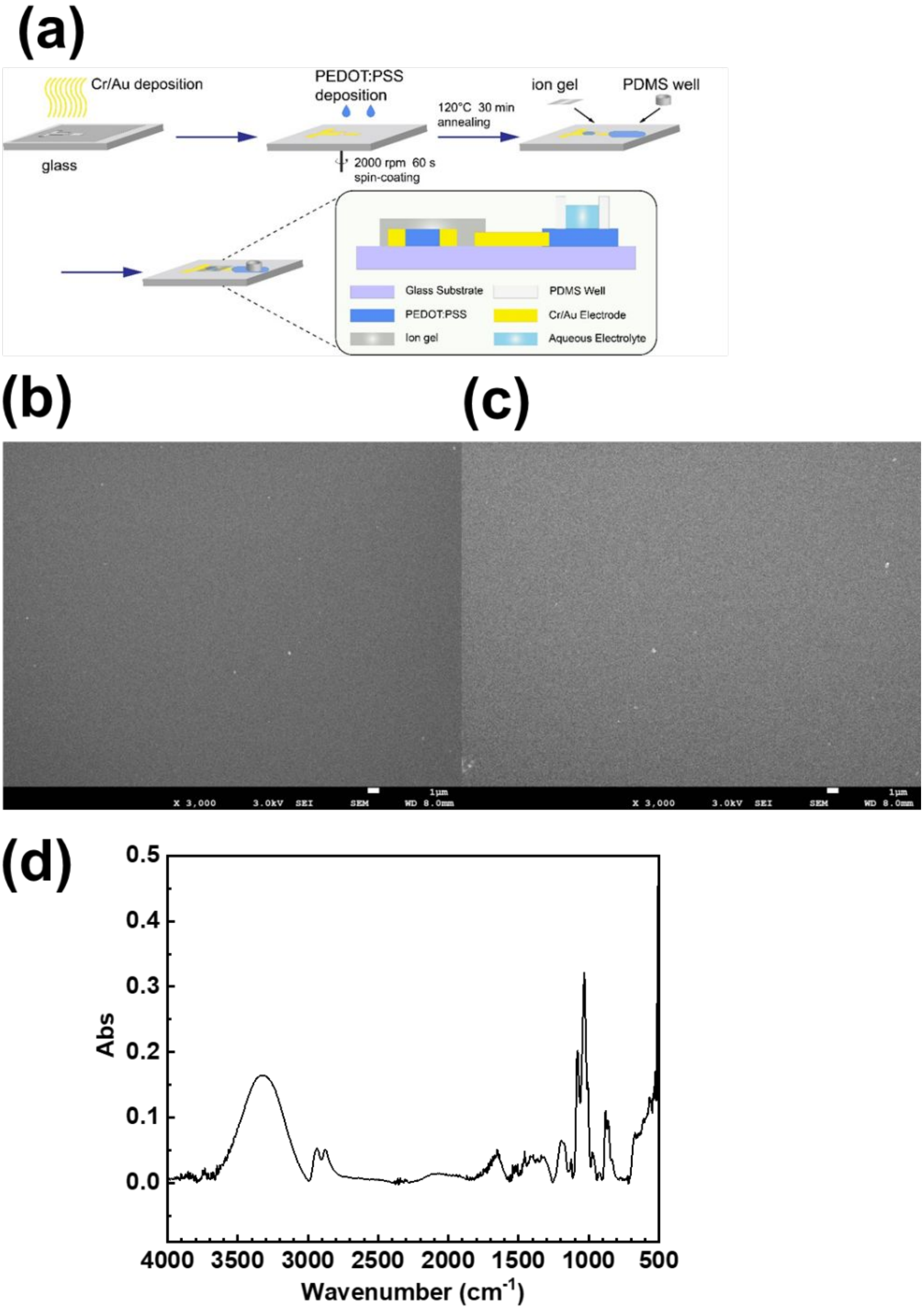

Figure S1. (a) Schematic illustration of fabricating the OECT-based artificial synaptic device with a floating-gated structure. (b)(c) SEM images of (b) the channel and (c) floating-gate PEDOT:PSS layers. (d) FT-IR spectrum of our PEDOT:PSS aqueous solution.

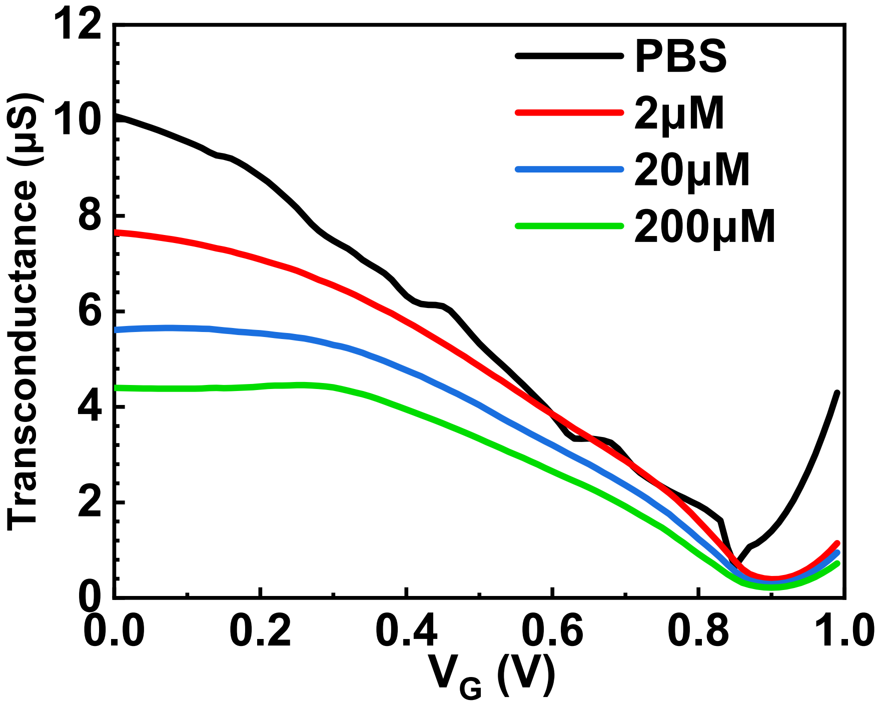

Figure S2. Transconductance characteristics measured in the aqueous electrolyte with different concentrations of dopamine.

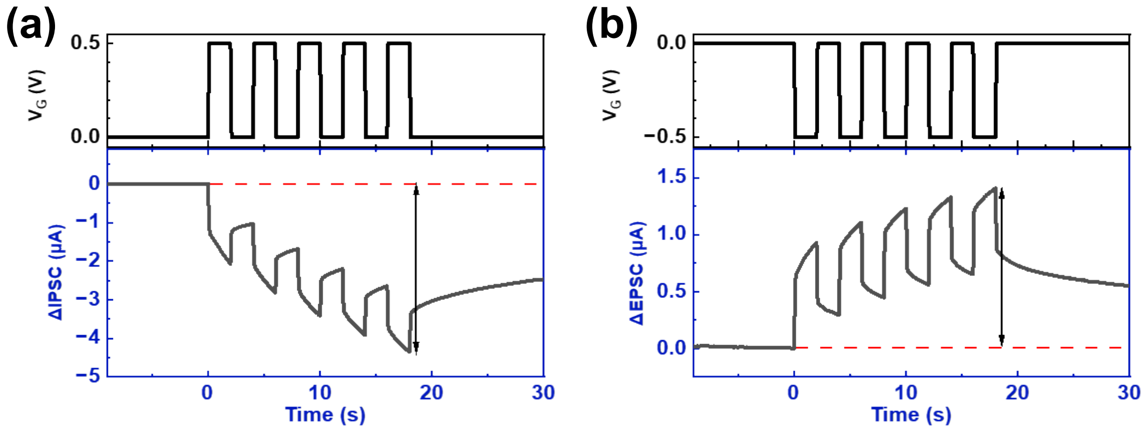

Figure S3. Synaptic response and memory effect (bottom) stimulated by applying five identical successive single voltage pulses ((a)V_G =+ 0.5 V,(b) V_G =－0.5 V, t = 2 s, top) to the gate electrode.

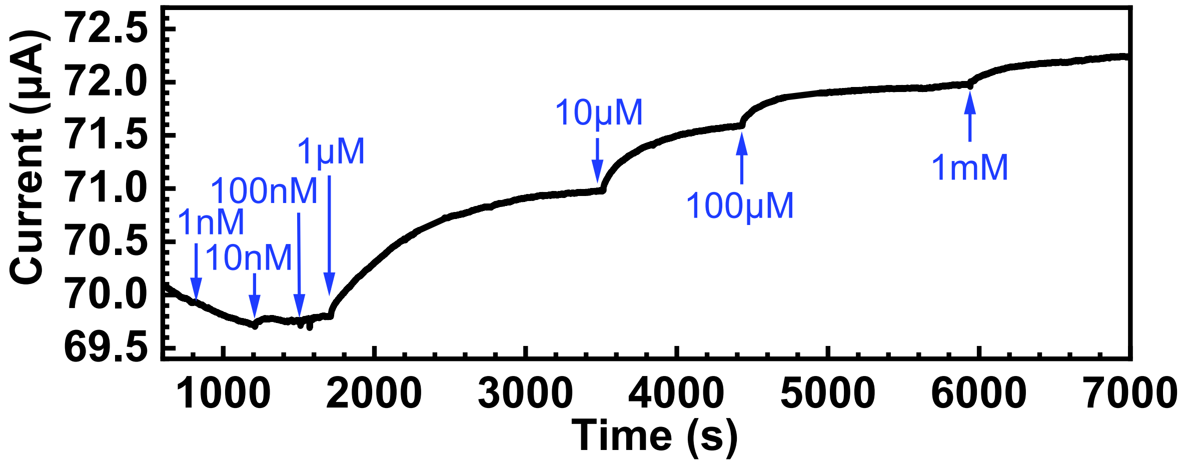

Figure S4. The real-time response of the device to the addition of 1 nM, 10 nM, 100nM, 1 μM, 10 μM, 100 μM, and 1 mM dopamine.

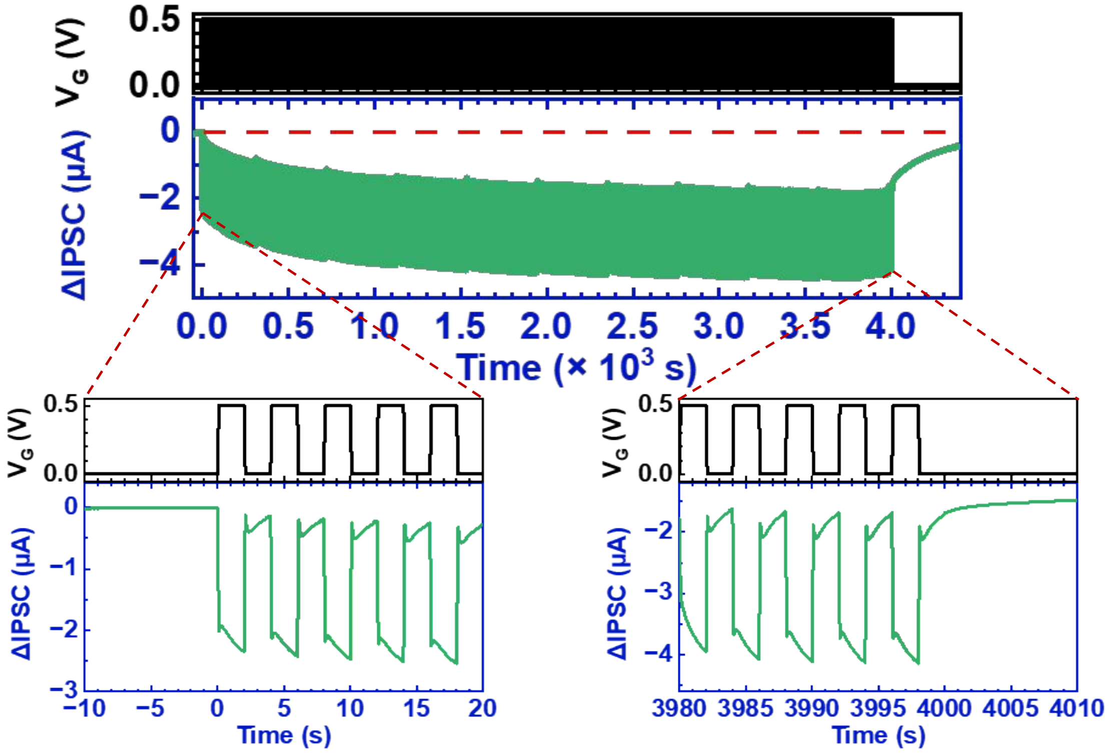

Figure S5. Continuous $\Delta IPSC$ response of the OECT-based synapse to 1000 successive positive voltage pulses ($V_G = +0.5\ V, t = 2\ s$) in the presence of 200 μM dopamine.

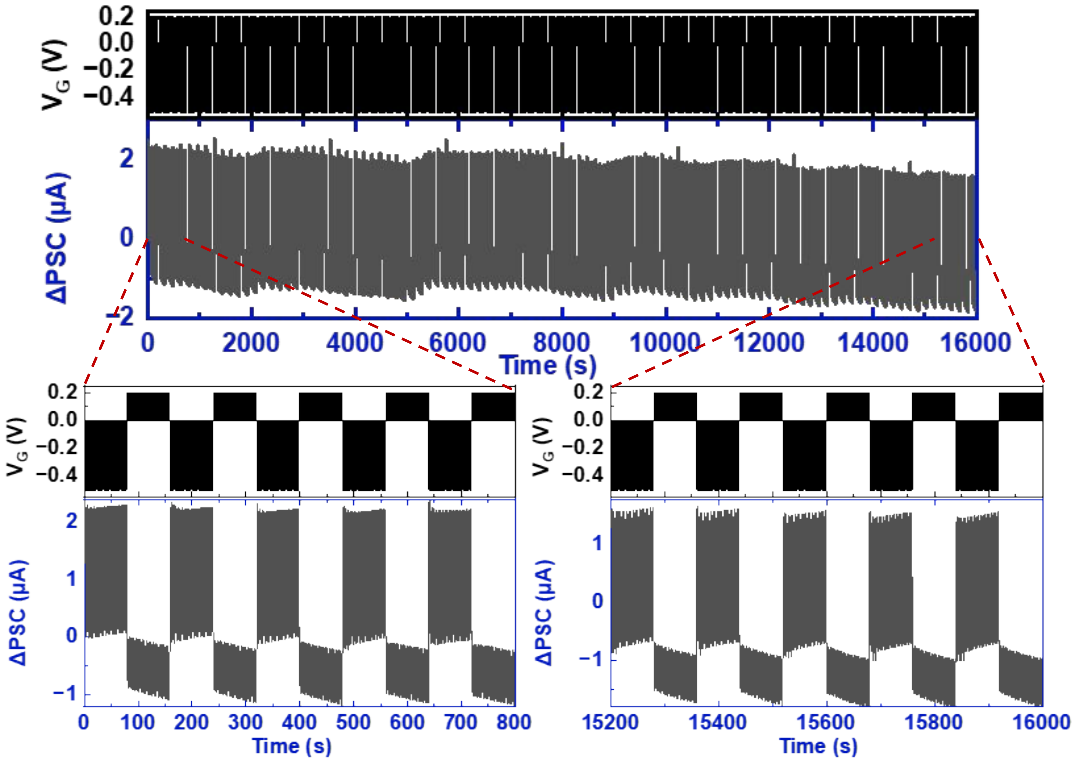

Figure S6. Long-term reliability test showing 100 consecutive cycles of the synaptic switching protocol (as described in Figure 5e).

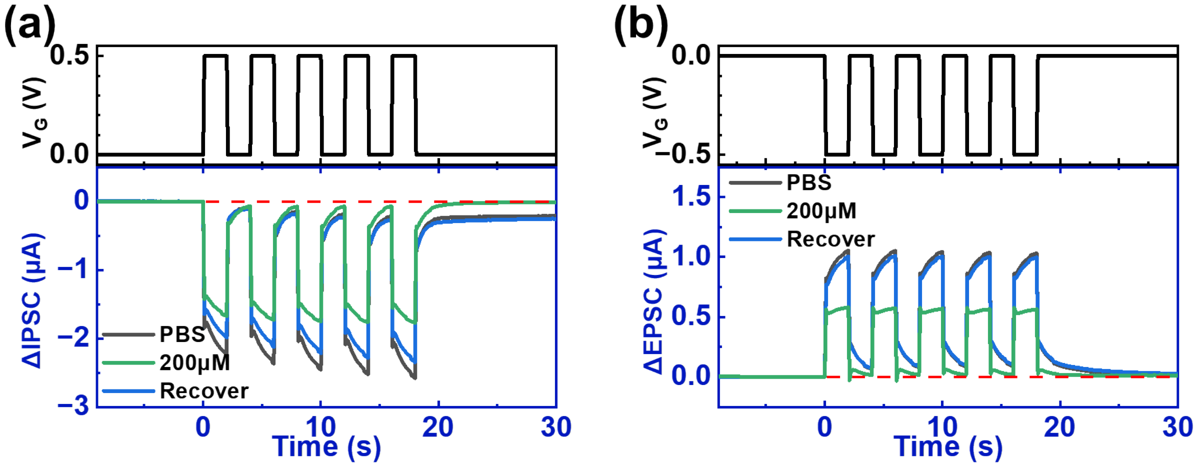

Figure S7. Comparison of the (a) ΔIPSC and (b) ΔEPSC modulation under three sequential conditions: initial PBS (black), 200μM dopamine (green), and after flushing the secondary electrolyte with fresh PBS (blue, labeled as "Recover"). The gate voltage pulses were V_G = +0.5 V for ΔIPSC and V_G = -0.5 V for ΔEPSC (t = 2 s).

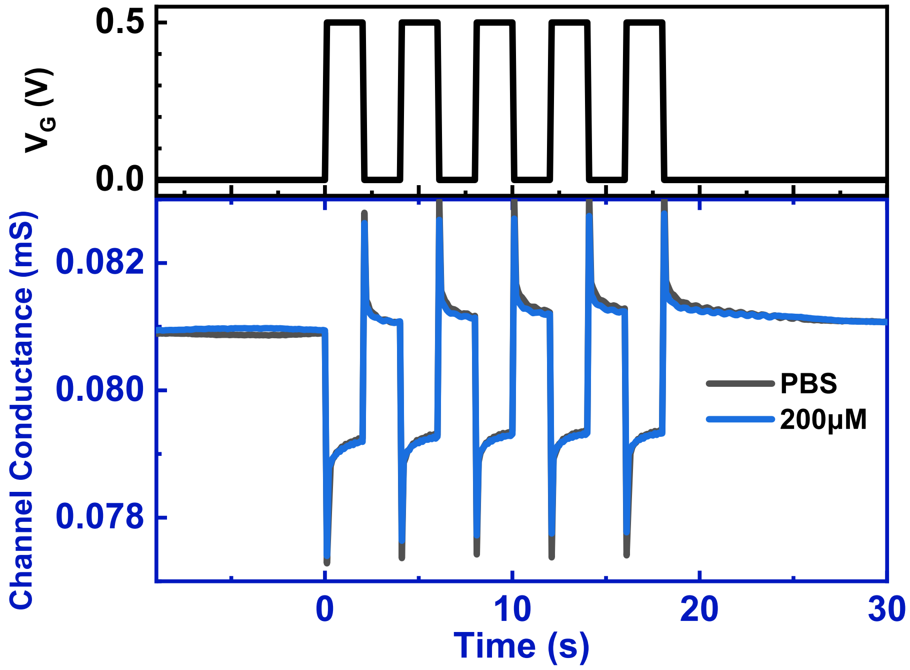

Figure S8. Synaptic behaviors and the memory effect of the device without PEDOT: PSS thin film in the floating gate (control device), without (bottom, black line) and with (bottom, blue line, concentration 200 μM) dopamine in aqueous electrolyte.

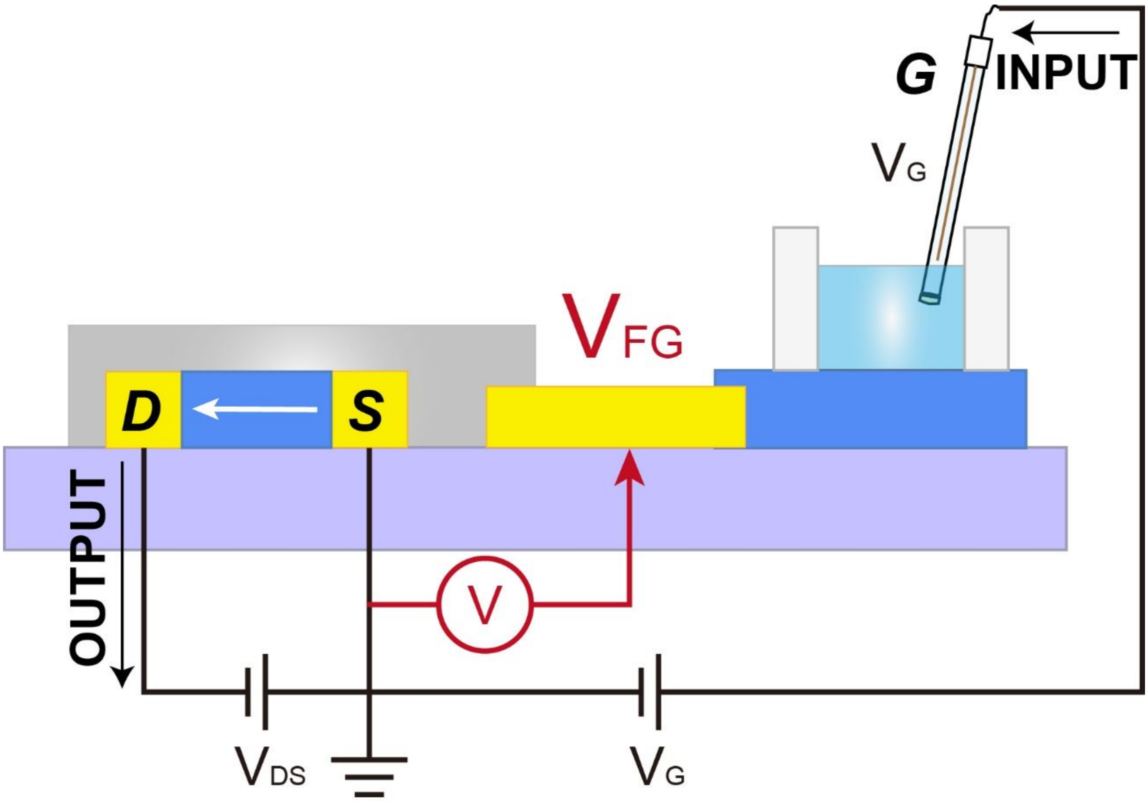

Figure S9. Schematic illustration of the probe and oscilloscope (red lines) to detect changes in the floating gate voltage (V_{FG}).

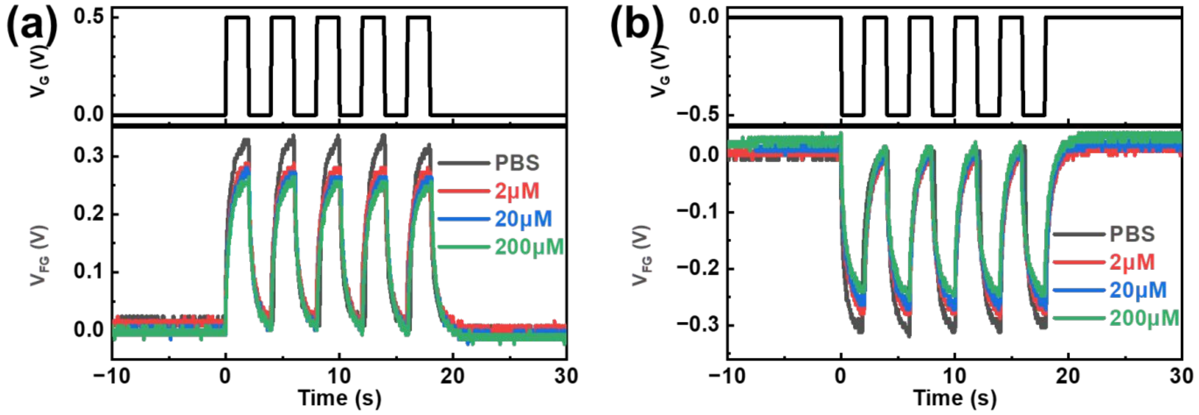

Figure S10. Floating gate voltage (V_{FG}) versus gate voltage (V_G) without (bottom, black line) and with (bottom, other lines, concentration 2, 20, and 200 μM) dopamine in the aqueous electrolyte. (a) positive gate pulses; (b) negative gate pulses.

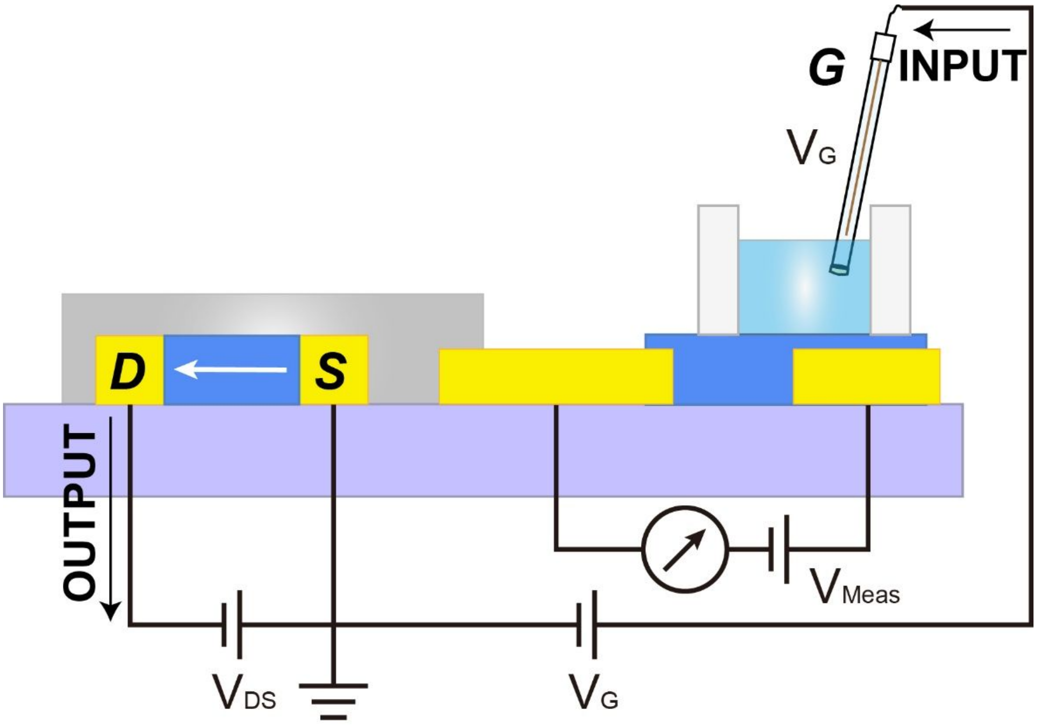

Figure S11. Schematic illustration of the electronic circuit to detect resistance changes of the floating-gate PEDOT:PSS.

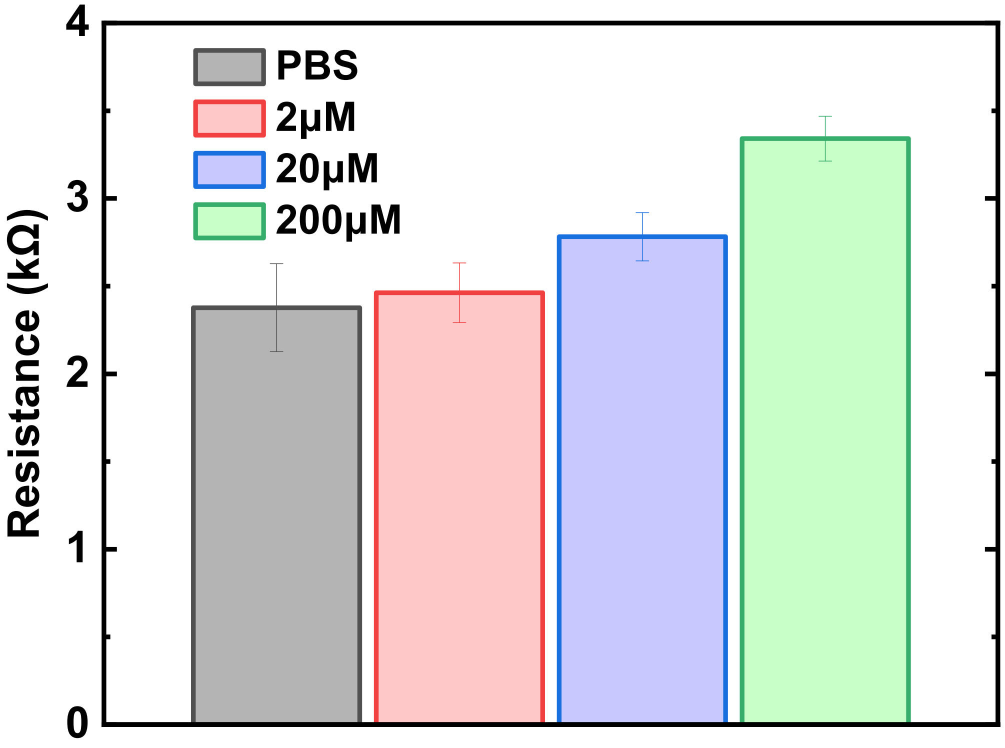

Figure S12. Comparison of the resistance changes of the floating-gate PEDOT:PSS at different dopamine concentrations.

References

[1] Bernards, D. A.; MacAya, D. J.; Nikolou, M.; Defranco, J. A.; Takamatsu, S.; Malliaras, G. G. *J. Mater. Chem.* **2008**, *18*, 116.

[2] Liao, C.; Mak, C.; Zhang, M.; Chan, H. L. W.; Yan, F. *Adv. Mater.* **2015**, *27*, 676.
